# Genetic variability and evolutionary dynamics of atypical *Papaya ringspot virus* infecting Papaya

**DOI:** 10.1371/journal.pone.0258298

**Published:** 2021-10-12

**Authors:** Anam Saleem, Zahid Ali, Shyi-Dong Yeh, Wajeeha Saeed, Amna Binat Imdad, Muhammad Faheem Akbar, Richard E. Goodman, Saadia Naseem

**Affiliations:** 1 Department of Biosciences, Plant Biotechnology and Molecular Pharming Lab, COMSATS University Islamabad (CUI), Islamabad, Pakistan; 2 Department of Plant Pathology, National Chung Hsing University, Taichung, Taiwan; 3 Department of Agriculture and Agribusiness Management, University of Karachi, Karachi, Pakistan; 4 Department of Food Science and Technology, University of Nebraska-Lincoln, Lincoln, NE, United States of America; National Cheng Kung University, TAIWAN

## Abstract

*Papaya ringspot virus* biotype-P is a detrimental pathogen of economically important papaya and cucurbits worldwide. The mutation prone feature of this virus perhaps accounts for its geographical dissemination. In this study, investigations of the atypical PRSV-P strain was conducted based on phylogenetic, recombination and genetic differentiation analyses considering of it’s likely spread across India and Bangladesh. Full length genomic sequences of 38 PRSV isolates and 35 CP gene sequences were subjected to recombination analysis. A total of 61 recombination events were detected in aligned complete PRSV genome sequences. 3 events were detected in complete genome of PRSV strain PK whereas one was in its CP gene sequence. The PRSV-PK appeared to be recombinant of a major parent from Bangladesh. However, the genetic differentiation based on full length genomic sequences revealed less frequent gene flow between virus PRSV-PK and the population from America, India, Colombia, other Asian Countries and Australia. Whereas, frequent gene flow exists between Pakistan and Bangladesh virus populations. These results provided evidence correlating geographical position and genetic distances. We speculate that the genetic variations and evolutionary dynamics of this virus may challenge the resistance developed in papaya against PRSV and give rise to virus lineage because of its atypical emergence where geographic spread is already occurring.

## Introduction

Plant viruses cause significant losses to the quantity and quality of most cultivated crop plants, bringing the greatest threat to their economic profitability. The real-time management of invading viruses requires accurate diagnosis which is now made possible through the advent of next generation sequencing (NGS) [1].This method enables the exploration of gene functions and contributes towards efficient disease management thereby avoiding subsequent crop losses and ensuring food security. However, the genetic diversity of virus populations is a major hurdle to devise befitting disease management strategies and must be addressed [1].

*Papaya ringspot virus* (PRSV) is a devastating pathogen for papaya and cucurbits production worldwide. The virus is transmitted by aphids in nature and belongs to the genus *Potyvirus*, family *Potyviridae* [2]. There are two serologically indistinguishable biotypes of PRSV which differ in their ability to infect papaya [3]; papaya-infecting biotype P (PRSV-P) which also infects cucurbits, and cucurbit-infecting biotype W (formerly known as *Water melon mosaic virus* 1) (PRSV-W) [4]. PRSV–P was first isolated from papaya in Hawaii [5]. Later on, it has been reported from several countries and is regarded as an extremely damaging pathogen within the initial years of its infection [4, 6]. Typical symptoms induced by the virus include the characteristic ringspots on fruits, along with mosaic, chlorosis, distortion on leaves, stunting in growth, flower abortion and water based oily streaks on petioles and upper young trunks. The PRSV possesses a single-stranded, positive sense RNA genome. The RNA is encapsidated with a coat protein of 36 KDa [7]. During favorable climatic conditions the virus leads to 100% yield losses [8]. The viral genome contains 10,326 nucleotides followed by a poly A tail and has a single open reading frame encoding a polyprotein of 3344 amino acids (nt 86–10120), which is processed by three viral proteases to generate all viral proteins [7].

The mutation, recombination, and reassortment are known to contribute towards genetic variation of potyviruses [9] as is the case with PRSV. The investigation of their role is important to reveal the evolutionary dynamics of this virus as well as understanding the conserved domains of the genome [10]. Past studies conducted on PRSV genome dynamics focused on getting insights into the adaptation of the virus to the changing environment. Preliminary evidence on the molecular evolution of PRSV suggested that PRSV-P might be originated from the type W, as PRSV-W appeared in Australia at least 20 years before the PRSV-P [3]. Studies on the CP sequences of most of the PRSV-P and some PRSV-W from different geographies have been conducted to estimate relative genetic divergence [3, 11]. The data based on the nucleotide and amino acid sequences of the CP genes of these isolates suggested a sequence divergence of up to 14% and 10% among these isolates [12, 13]. However, sequence analysis of the CP genes from the USA and Australia showed only marginal variation in their PRSV isolates [11, 12]. The CP gene sequences of PRSV isolates from Pakistan and geographic locations from five continents had shown that isolates from Pakistan emerging as atypical virus and evolutionarily diverse in its coat protein (CP) structure [14, 15].

Recently, the transgenic protection has taken over conventional methods to manage PRSV which yielded low success rates [16, 17]. But the variation of PRSV associated with the geographical locations and homology-dependent expression of transgenic resistance limit its effectiveness [18]. In case of papaya, the viral suppressor gene HC-Pro, of PRSV contributes to the decline of transgenic resistance [19]. The strong silencing suppressor gene HC-Pro has strong affinity towards secondary siRNAs, which clings to other heterologous viruses and collectively support PRSV invasion [19, 20]. Considering the fact that high diversity is associated with PRSV populations, their genetic differences may limit global disease management [21]. Thus a deeper understanding of sequence divergence and evolutionary dynamics of genes is required to understand potential breakdown of CP transgenic resistance [22]. The genomic variability associated with HC-Pro and the N-terminal region of the functionally important PRSV-P1 gene has already been discussed in detail [10], therefore genome dynamics of these genes must also be addressed in context of Pakistan.

The existing literature mostly covers the genetic diversity studies based on individual gene fragments of PRSV particularly the CP gene [7, 11–13, 15]. Several recent studies described evolutionary basis of PRSV through comparative analysis using the whole genome [23–25]. Before this study, the evolutionary nature of the PRSV-PK strain at the whole genome level has not been explored. Also, there is ambiguity about earliest identification of virus from cucurbits. Therefore, present study was designed to investigate the PRSV-PK strain in depth and to reveal the insight of PRSV-PK genome through comparative genomic analysis with the PRSV isolates from other Asian and American countries. The comparison of the annotated whole genome sequence of PRSV-PK, and the fragments including PI, CP, HC-Pro and 3’ UTR with representative genome segments of PRSV isolates from rest of the world has been investigated. The derived phylogenetic relationships are helpful to explore the spread, interlinking and complexity of PRSV populations worldwide. The current work makes its valuable contribution in formulation of strategies for effective strain management at regional level. It has further implications in improving food security and related economic benefits.

## Materials and methods

### PRSV source

The Papaya growing regions in Sindh province of Pakistan were surveyed from 2011–2018. Random sampling was done from six districts including Malir district (24.8937° N, 67.2163° E), Tando Adam (25.7682° N, 68.6559° E), Tando Allahayar (25.4570° N, 68.7215° E), Tandojam (25.4281° N, 68.5307° E), Tando Muhammad Khan (25.1256° N, 68.5426° E) and Sakkhar (27.7244°N, 68.8228°E). PRSV leaf samples showed typical symptoms of mosaic, leaf distortion and mottling were collected. The samples, nucleic acids and lyophilized infectious material were stored at –80°C for further processing. Samples were processed and evaluated by ELISA and RT–PCR following the protocol described earlier [15]. The virus was found in all six regions and after molecular characterization were designated as PRSV-PK isolates.

### Virus recovery and purification

The plants of *Cucumis metuliferous*, *Chenopodium quinoa* and *Carica papaya* cv. Tainung 2 were mechanically inoculated with the inoculum, prepared by grinding 500 mg of PRSV infected leaf tissue of papaya in 5 mL phosphate buffer 0.05 M, pH 7.0 using pestle and mortar. The inoculated plants were kept in the glasshouse (28/22°C day/night). The typical local lesions were induced on *C*. *quinoa* upon inoculation by the respective inoculum. The local lesions were subsequently transferred to *C*. *quinoa* at least three times aiming to purify the virus. The inoculum was finally inoculated on *C*. *papaya* and *C*. *metuliferous* to maintain PRSV–PK in these propagative hosts. The virus purification assay was performed with five biological and three technical replicates. Healthy plants of *C*. *metulifreous*, *C*. *papaya* and *C*. *quinoa* were kept as a control.

#### RT–PCR, cloning and genome sequencing of PRSV

Total RNA was extracted from leaves of the *C*. *papaya* plants infected with PRSV-PK using Trizol Reagent (Ambion, Life technologies). The cDNA was synthesized using Oligo dT (18) primer, 2.5 mM dNTPs with M-MLV reverse transcriptase (Invitrogen) and 1X reaction buffer, MgCl_2_, dTT and RNase block, the mixture was incubated at 42°C for 1 hour followed by 10 min at 70°C. The Highthroughput sequencing (HTS) was performed on Illumina platform and subsequent custom bioinformatic analysis for contig alignment to the PRSV viral genome. The draft genome sequence was searched for prediction of coding sequence (CDS) by using BLAST. To confirm the sequence draft subsequent amplification and sequencing were performed as detailed. KOD-Plus Taq polymerase (TOYOBO, China) and PRSV specific primers (S1 Table) were used at specific T_m_ and optimized PCR profiles. The amplified fragments were analyzed in 1% agarose gel in Tris Borate EDTA (TBE) buffer. The gel was visualized under UV transilluminator and the estimation of product size was made with 10 Kb ladder (Invitrogen, USA). The PCR products were purified with Gene Mark (GMbiolab Co., Ltd., Taichung, Taiwan) PCR purification Kit and cloned in Topo Blunt II vector (Invitrogen, San Diego, CA, USA) using 1 unit enzyme/1 μg DNA as per manufacturer’s protocol. Cloned plasmids were purified through Gene mark Plasmid Purification Kit (GMbiolab Co., *Ltd*., Taichung, Taiwan) and confirmed for insert through M13 Forward and M13 reverse primer amplification. The plasmids were subsequently sequenced. The ends of the virus genome were determined by RACE [26].

**Fig 1:**
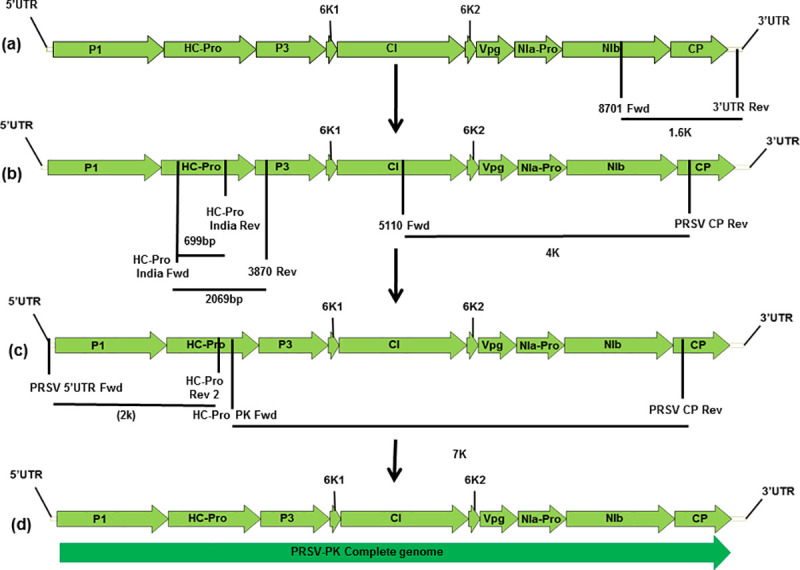
Schematic amplification of PRSV–PK genome. (a) CP gene amplification; (b) 4K, HC-Pro and full HC–Pro amplification; (c) 5’ terminal end and 7K amplification; (d) Full genome amplification. Open reading frames are indicated as arrows on viral sense RNA. The direction of arrow indicates the direction of translation. PI;HC-Pro; P3:; 6K1; CI: Cylindrical inclusions; 6K2; Vpg; NIa-Pro; NIb; CP: coat protein.

Full length CP gene was amplified using the primer pairs 8701 Fwd and 3’ UTR Rev (S1 Table) with an amplicon size of about 1.5 Kb. The sequencing of CP fragment and BLAST analysis enabled to design primer for subsequent amplification. The next amplicon was generated with forward primer from the middle of CIP and one internal CP reverse primer. PCR was performed using KOD polymerase with 94°C initial denaturation for 2 min, 30 cycles of 15 s melting at 94°C, 15 s annealing at 54°C, and 3 min 50 sec extension at 68°C; with a final extension at 68°C for 10 min. Fig 1 shows the illustration of the amplified fragment. For amplification of rest of the genome random primers forward and reverse, sequences mentioned in S1 Table, were designed to amplify the segment within HC-Pro with an amplicon of 799 bp. The predetermined sequence of HC-Pro gene was used to design specific primers to amplify the 5’ genomic RNA of PRSV-P isolate PK by using 5’UTR forward paired with HC-Pro Rev 2. The final amplification was performed using the Primers HC-Pro forward coupled with PRSV-PK internal CP reverse which yields a 7 Kb fragment to complete the genome sequence. The product was directly sequenced via nanopore sequencing. All primer sequences used in this study are listed in the S1 Table and schematic amplification illustration for complete PRSV-P isolate PK is shown in the (Fig 1).

### Phylogenetic analysis of PRSV-PK genome

The evolutionary history of PRSV has been inferred with the use of the Maximum likelihood method for phylogenetic analysis in MEGAX [27]. In this phylogeographical analysis complete genomes, coat protein sequences, P1, HC-Pro coding sequences, and highly conserved 3’UTR domain sequences of PRSV were included in the database. Alignment of nucleotide sequences were done using ClustalX2. The evolutionary distance has been calculated as arm length in the tree using Tamura-Nei model A bootstrap test of 1,000 replicates was used to determine the phylogenetic tree [28]. Neighbor-Joining method was also used to infer the evolutionary history of PRSV. The evolutionary distance has been calculated as arm length in the tree using Maximum-Composite likelihood method.

Full length individual coat protein gene sequences of PRSV isolates from Pakistan were compared with 43 differential PRSV CP sequences from America, China, Taiwan, India, Bangladesh, Vietnam, Mexico, Japan, Malaysia, Philippines, Thailand, Ecuador, Australia and Colombia obtained from GenBank database. Similarly, for the genome sequence analysis of P1, HC-Pro and 3’UTR regions, sequences were retrieved from full length genome accessions of GenBank; aligned with the individual sequence of atypical PRSV-PK strain and phylogeographic analysis was conducted. For the PRSV complete genome sequence analysis in this study, we have used representative PRSV-PK sequence and compared it with full length genome sequences of 37 PRSV isolates from multiple geographies retrieved from the GenBank database on the basis of BLAST search. The sequences of isolates from America, China, Taiwan, Thailand, Ecuador, New Guinea, India and Colombia were retrieved during the study. Recently sequenced PRSV isolates from West Bengal-India [25] while BD2 and BD1 from Bangladesh [24] have been included phylogenetic as well as subsequent analysis. *Sugarcane mosaic virus* (SCMV), strain Babati10 TZA-Tanzania with accession no. MN795295 has been used as outgroup during phylogenetic analysis.

**Fig 2:**
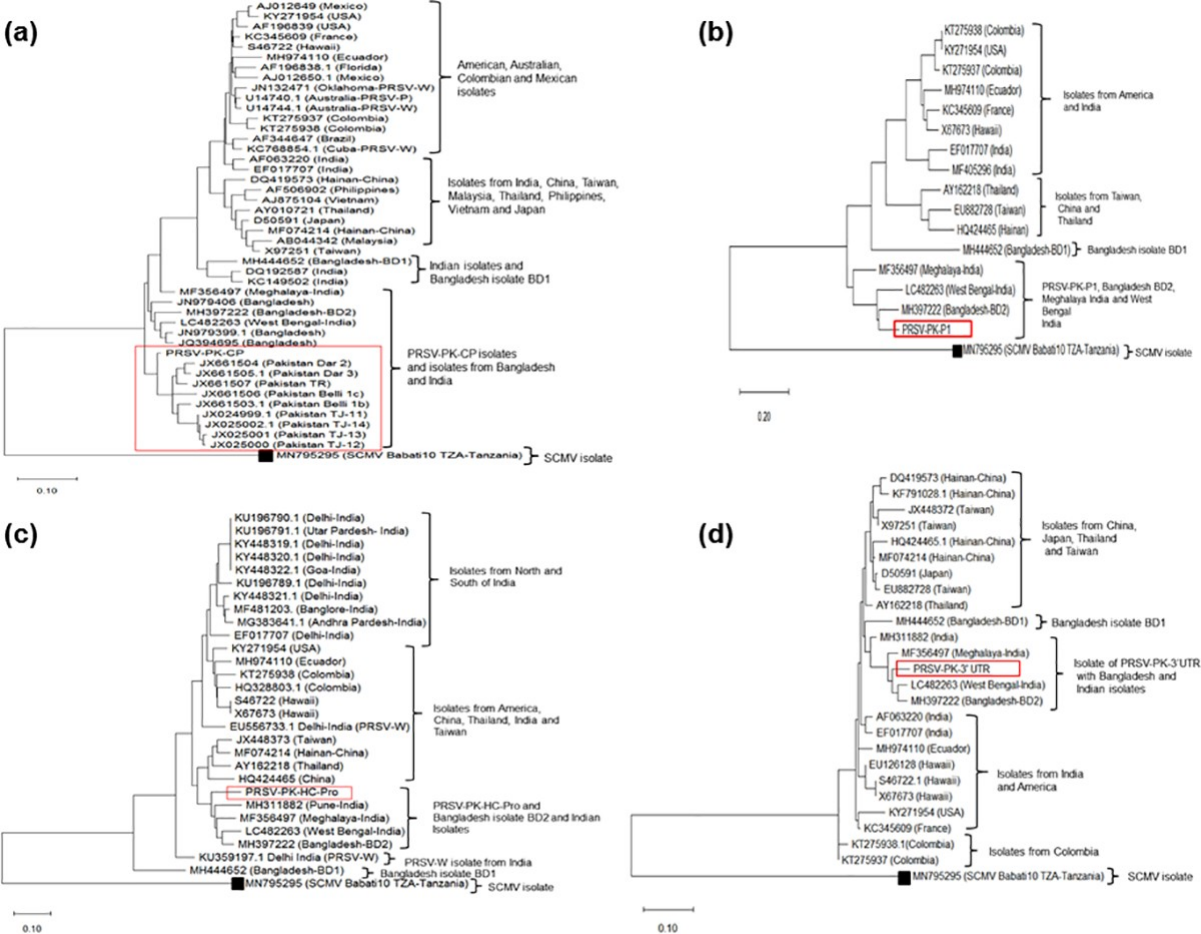
Maximum likelihood tree representing phylogenetic relationships of *Papaya ringspot virus*-P strain PK (PRSV–PK). (a) Coat Protein (CP) sequence (b) Protease P1 sequence (c) Helper component HC-Pro Sequence (d) 3’UTR sequence from other related sequences selected via BLAST search. Corresponding CP, P1, HC-Pro and 3’UTR sequences of *Sugarcane mosaic virus* (SCMV) (accession no. MN795295) used as outgroup. Upper and lower branch points show bootstrap values (1,000 replicates) supporting a particular phylogenetic group. The scale bar represents nucleotide substitutions per site. All nucleotide sequences are retrieved according to the isolate name and the GenBank accession number.

The sources of CP, P1, HC-Pro, 3’UTR and whole genome sequences of PRSV isolate of Pakistan and other countries used in this study are shown in S2 Table. The phylogenetic reconstruction has been documented as trees (Fig 2). Differential CP gene sequences of the nine PRSV Pakistani isolates are available in GenBank (accession numbers; JX025001, JX025000, JX025002, JX024999, JX661503, JX661506, JX661507, JX661504, JX661505) [15]. The complete genome sequence of PRSV-P isolate PK (MT090406) has been submitted from this study.

### Genetic differentiation, Gene flow and recombination analysis

The DnaSP 6.12.03 program [29] was used to analyze genetic differentiation and the level of gene flow between the populations of PRSV-PK based on CP, P1, HC-Pro and complete genome sequences with PRSV populations from various geographical areas with the use of statistic Kst* [30]. Another test statistic Snn [31] was used to measure the frequency of the nearest neighbor sequences in the same locality, whose values may range from 1 (when populations from different localities are genetically distinct) to 1/2 in the case of substantial equal populations from equivalent populations [31]. The analysis was performed from tests of 1,000 replicates. The gene flow assessment between PRSV population based on CP, P1 and HC-Pro as well as full genome was calculated using Fst [32]. Fst value (fixation index) indicating the amount of inter-population diversity and the value ranges from zero (indicating no differentiation between populations) to one (indicating full differentiation between populations) [30]. The coefficient of Fst estimates the extent of inter-population genetic differentiation [33]. An absolute value of Fst > 0.33 suggests gene flow is negligible, while absolute value of Fst < 0.33 indicates gene flow is frequent [34].

PRSV Coat protein gene sequences of 44 isolates including Pakistan (n = 10), Bangladesh (n = 5), India (n = 6), Australia (n = 2), America (n = 9), Mexico (n = 2), other Asian countries (China, Taiwan, Japan, Thailand, Vietnam, Malaysia and Philippines) (n = 8) and Colombia (n = 2) were divided into net number of 8 population. Full length PRSV genome sequences of 39 isolates were grouped into a net number of 7 Population comprising Pakistan (n = 2), India (n = 10), Colombia (n = 2), America (n = 6), New Guinea (n = 4), Bangladesh (n = 2) and other Asian countries (China, Taiwan and Thailand) (n = 13) and analyzed for gene flow assessment. The PRSV population from Pakistan was compared with the rest of the 6 populations individually. Full length P1 sequences of 17 PRSV isolates, from Pakistan (n = 2), Other Asian countries (Thailand, China and Taiwan) (n = 3), India (n = 4), America (n = 4), Bangladesh (n = 2) and Colombia (n = 2) were analyzed for gene flow and genetic differentiation analysis. Moreover, gene flow and genetic differentiation based on HC-Pro sequences of PRSV isolates were also performed. PRSV-PK-HC-Pro population was compared with the rest of 5 populations from, India (n = 15), Colombia (n = 2), Bangladesh (n = 2), America (n = 4) and Other Asia (China, Taiwan, Thailand) (n = 4). Sites with alignment gaps were excluded. After defining populations, the genetic differentiation and gene flow was analyzed using DnaSP6 [29].

Recombination evidence in the genome sequences of PRSV was searched with the program RDPv4.97 software package [35] that implements a range of recombination-detecting algorithms including GENECONV [36] BOOTSCAN [37, 38], MAXCHI [39, 40], CHIMAERA [40], SISCAN [41], 3SEQ [42], and RDP [43]. In RDP analysis, every combination of three sequences in the input alignment is sequentially tested to demonstrate two sequences as a parent and third as a recombinant. The analysis of recombination was performed, between and within groups (i.e. countries) of sequences and the events detected by at least three different algorithms were accepted as evidence (breakpoint) of recombination. The recombination effect was considered diligently during selection analysis. The possible recombination breakpoints were also detected in the particular event of each RDP method.

The recombination analysis was performed to check the recombination signals in the core CP region. RDP was also used to check recombination signals in the aligned full length genome of PRSV-PK and 37 reported complete PRSV genome sequences from Hawaii, Taiwan, France, USA, Colombia, India, China, Bangladesh, New Guinea, Ecuador and Thailand.

## Results

### Sequence variability and phylogenetic assessment of CP, P1, HC-Pro, 3’UTR and PRSV whole genome

The verified whole genome sequence of atypical PRSV-P isolate PK has been drafted through sequencing and analysis with aligned global PRSV sequences. The detail on cloning of amplification fragments in its genome is schematically presented in Fig 1 (Fig 1); annotated genome sequence has been submitted in GenBank (Accession number MT090406). The sequence analysis using CP, P1, HC-Pro, 3’UTR gene fragments and the resulting phylogenetic tree derived from the comparison of these individual sequences is shown in the Fig 2A–2D respectively (Fig 2). The highly variable complete CP gene nucleotide sequence of this virus made an independent cluster with former sequences of PRSV-CP gene originated from Pakistan, confirming the genetic variants of the same strain of the virus. The closest relatives become the previously sequenced CP isolates of Pakistan. The extended similarity of it becomes with JQ394695 and JN979399 from Bangladesh with 92.8% homology and LC482263 a recently reported isolate from West Bengal-India with 92.4% homology. The overall clade extended further to two isolates from Bangladesh JN979406; MH397222-BD2and an Indian isolate from Meghalaya MF356497 in the geographic region (Fig 2A). The CP gene sequences of PRSV strains from America, China, Vietnam, Australia, Thailand, Japan, Malaysia, Philippines, Taiwan and rest of India are included in a large separate clade, showed least homology with our representative virus.

The P1 gene of PRSV-PK possessed high sequence variation mainly because of nucleotide and subsequent amino acid insertions. The comparative phylogenetic analysis of PRSV-PK-P1 gene with PRSV isolates from diverse geographical locations showed highest homology (85.7%) with BD2 isolate, followed by 83.46% and 81.0% homology with isolates from West-Bengal and Meghalaya.

This phylogenetic tree (Fig 2B) illustrates the genome variation of PRSV-PK with the American and other Asian isolates. The phylogenetic analysis of the P1 gene generated three clades, first having PRSV-PK along with Bangladesh isolate BD2 -MH397222 along with the Indian isolate from West Bengal- LC482263and another Indian isolate Meghalaya-MF356497. Whereas, isolates of USA, Colombia, Hawaii, Ecuador (South America), India and France origin were grouped in a separate cluster. Yet again another cluster consisted of PRSV isolates from other Asian countries (Thailand, Taiwan and Hainan-China) (Fig 2B).

Another, variable region in PRSV genome is HC-Pro which was also analyzed for genome variability. The HC-Pro gene of PRSV-PK formed a separate clade with three Indian isolates, from Meghalaya, Pune, West Bengal and BD2 isolate from Bangladesh. The highest homology for this gene was 91.5%with BD-2 isolate. The phylogenetic tree showed isolates from southwestern and many isolates from northern regions of India as divergent (Fig 2C). The degree of relatedness of 3’UTR assessed through phylogenetic tree depicts that the 3’UTR of PRSV–PK is closely related to the PRSV isolate from Meghalaya (MF356497) (93.6%) and Bangladesh-BD2 (MH397222) (93.6%). Whereas, the similarity of this clade extends to the two Indian isolates, LC482263 (93.6%) from West-Bengal and MH311882 (91.3%) from Pune; a region in west of India. Yet, the isolates from America, France, Colombia, India, Bangladesh, Ecuador, Thailand and few isolates from Taiwan showed substantial sequence variation from PRSV-PK. Still another divergent clade comprised majority of the isolates from China and Taiwan (Fig 2D).

**Fig 3:**
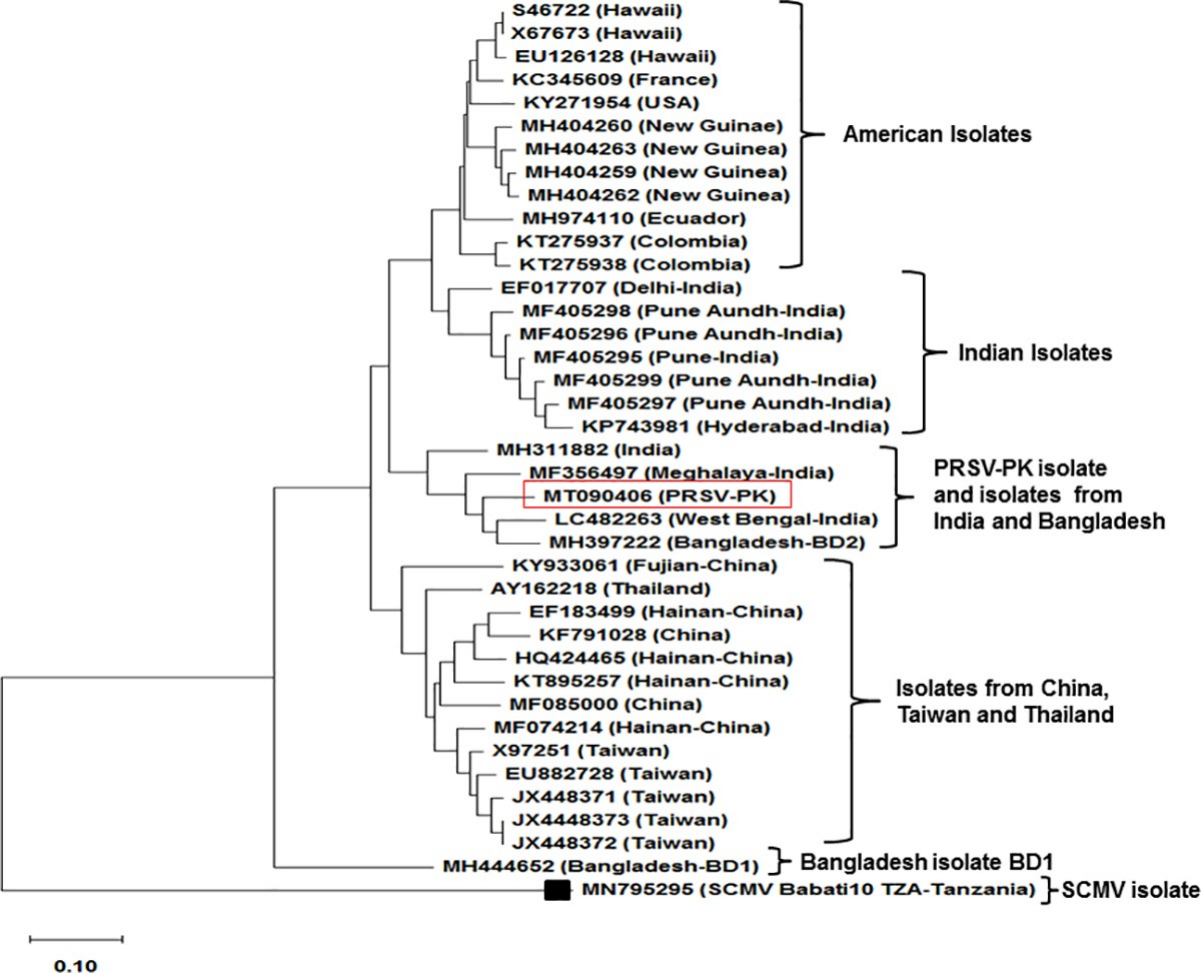
Region-specific maximum-likelihood tree representing the phylogenetic relationship between complete genome sequences of PRSV-P isolate of Pakistan and PRSV complete genome sequence from rest of the world. *Sugarcane mosaic virus* (SCMV) (accession no. MN795295) used as an outgroup. The tree was constructed using ClustalX2 and MegaX Program. The scale bar represents nucleotide substitution per site, the bootstrap value of 1,000.

The comparative phylogeographical occurrence of PRSV-PK has been demonstrated through conclusive tree construction, based on whole-genome sequences of PRSV from different geographical regions. It has demonstrated convincing overall tree topology results. The complete genome sequences of isolates with genome length up to 10,343 bp from China, Taiwan, Hawaii, USA, Colombia, Thailand, India, Bangladesh, Ecuador (South America), New-Guinea and France were retrieved from the NCBI database. The phylogenetic tree revealed that the full length PRSV-PK isolate (in this study) showed close homology of (89.8%) with BD2 isolate from Bangladesh. Other close relatives in the same clade include the isolates from West-Bengal, Meghalaya and Pune-India with 89.2%, 88% and 85%homology (Fig 3) respectively. The phylogenetic tree using NJ method based on individual fragments a) CP, b) P1, c) HC-Pro d) 3’UTR conclusively mentioned in S1 Fig. The tree based on whole genome constructed using NJ method has been mentioned in S2 Fig.

### Genetic differentiation and gene flow assessment in PRSV-PK genome

Three statistical parameters were used to estimate genetic differentiation between the PRSV-PK population and PRSV population from other geographical regions based on their CP, P1, HC-Pro and full genomic sequences. In the individually aligned CP, P1, HC-Pro and full-length genomic sequences, a permutation-based statistical test, Kst* (sequence-based statistical test) and Snn (measurement of the frequency of the nearest neighbor sequences in the same locality) were utilized to measure the genetic differentiation between PRSV population of Pakistan with the population from diverse geographical locations. The genetic differentiation estimation between the subpopulations using CP sequences created in DnaSP showed significant P values of Kst* and Snn (Table 1), which significantly denied the null hypothesis statement that “there is no genetic differentiation between two subtypes” thereby declaring genetic differentiation. Fst (the interpopulation component of genetic variation or the standardized variance in allele frequencies across populations) was used to access the gene flow level. Two subpopulations were created to compare the differences. Considering the data (Table 1) the Fst values generated between the populations based on the PRSV-CP isolates were above 0.33 except the values between populations of PK vs Bangladesh (0.26720) and PK vs India (0.32051). The descending order of Fst values between different populations includes PK vs Australian (0.75410), PK vs Colombian (0.7502), PK vs American (0.64828), PK vs other Asian isolates (China, Taiwan and Thailand) (0.61615), and PK vs Mexican (0.61422), depicts that there exists a significant genetic differentiation between these populations. The estimation of genetic differentiation and gene flow between populations using CP gene sequences are shown in Table 1.

**Table 1:** Statistical tests for genetic differentiation and gene flow between *Papaya ringspot virus* populations from Pakistan with the populations from Colombia, Mexico, America, Other Asian regions, Bangladesh, Australia and India based on the CP gene nucleotide sequences.

Populations	Kst*	P value	Snn	P value	Fst
Pakistan vs Colombian	0.16375	0.0020**	1.00000	0.0280*	0.7502
Pakistan vs Mexican	0.16149	0.0010**	1.00000	0.0290*	0.61422
Pakistan vs American	0.18293	0.0000***	1.00000	0.0000***	0.64828
Pakistan vs other Asian (Thailand, Taiwan, China, Veitnam, Malaysia, Japan, Phillipines)	0.17185	0.0000***	1.00000	0.0000***	0.61615
Pakistan vs Bangladesh	0.10152	0.000***	0.86667	0.01*	0.26720
Pakistan vs Australian	0.15728	0.0130*	1.00000	0.0330*	0.75410
Pakistan vs Indian	0.11121	0.0000***	0.87500	0.0070**	0.32051

ns, not significant

*0.01 < P < 0.05

**0.001 < P < 0.01

***P < 0.001.

Kst*, *Snn and Fst* were implemented in DnaSP 6. The deviation hypothesis from null population differentiation was tested by 1000 permutations of the raw data.

The descending order of Fst values calculated between PRSV-PK virus population with rest of 6 populations form different geographical regions based on full genomes includes, PK vs Colombia (0.90634), PK vs New-Guinea (0.89815), PK vs America (0.81973), PK vs Other Asian regions (0.71247), PK vs India (0.62259) and PK vs Bangladesh (0.26884). The estimation of genetic differentiation and gene flow between PRSV-PK populations with rest of the populations based on full genome sequences is mentioned in Table 2.

**Table 2:** Statistical tests for genetic differentiation and gene flow between *Papaya ringspot virus* populations from Pakistan with the populations from Colombia, Mexico, America, other Asian regions, Bangladesh, New Guinea and India based on the full-length genomic sequences.

Populations	Kst*	P-value	Snn	P-Value	Fst
Pakistan vs Indian	0.01198	0.0920^ns^	0.83333	0.2390^ns^	0.62259
Pakistan vs American	0.11261	0.0500^ns^	1.00000	0.0500^ns^	0.81973
Pakistan vs other Asian	0.01958	0.0000***	1.00000	0.0070**	0.71247
Pakistan vs Colombian	1.00000	1.0000^ns^	1.00000	0.3260^ns^	0.90634
Pakistan vs New Guinea	0.11705	0.0480*	1.00000	0.0530^ns^	0.89815
Pakistan vs Bangladesh	1.00000	1.0000^ns^	0.50000	0.3140^ns^	0.26884

ns, not significant

*0.01 < P < 0.05

**0.001 < P < 0.01

***P < 0.001

Kst*, *Snn and Fst* were implemented in DnaSP 6. The deviation hypothesis from null population differentiation was tested by 1000 permutations of the raw data.

The Fst values from highest to the lowest, indicates degree of gene flow between PRSV-PK-P1 with the rest of the population were 0.92427 (Colombia), 0.80487 (America), 0.7480 (other Asia), 0.46883 (India) and 0.27654 (Bangladesh) (S3 Table). The lowest Fst value recorded in case of PK vs Bangladesh depicts frequent gene flow between these populations. Similarly, the P–values of Kst* and Snn calculated between PRSV–Populations compared on the basis of HC-Pro gene depicts that populations are not well-differentiated (S4 Table). The highest to lowest Fst values between PRSV-PK-HC-Pro population compared with rest of population were 0.79116 (America), 0.73479 (Colombia), 0.67450 (other Asian), 0.59388 (India) and 0.32770 (Bangladesh) (S4 Table). The lowest Fst value was recorded in case of PK vs Bangladesh showing gene flow is not as infrequent as observed between other populations. The value of Fst >0.3 depicted that genetic differentiation still resides between Pakistan and Bangladesh populations.

### Recombination analysis

**Fig 4:**
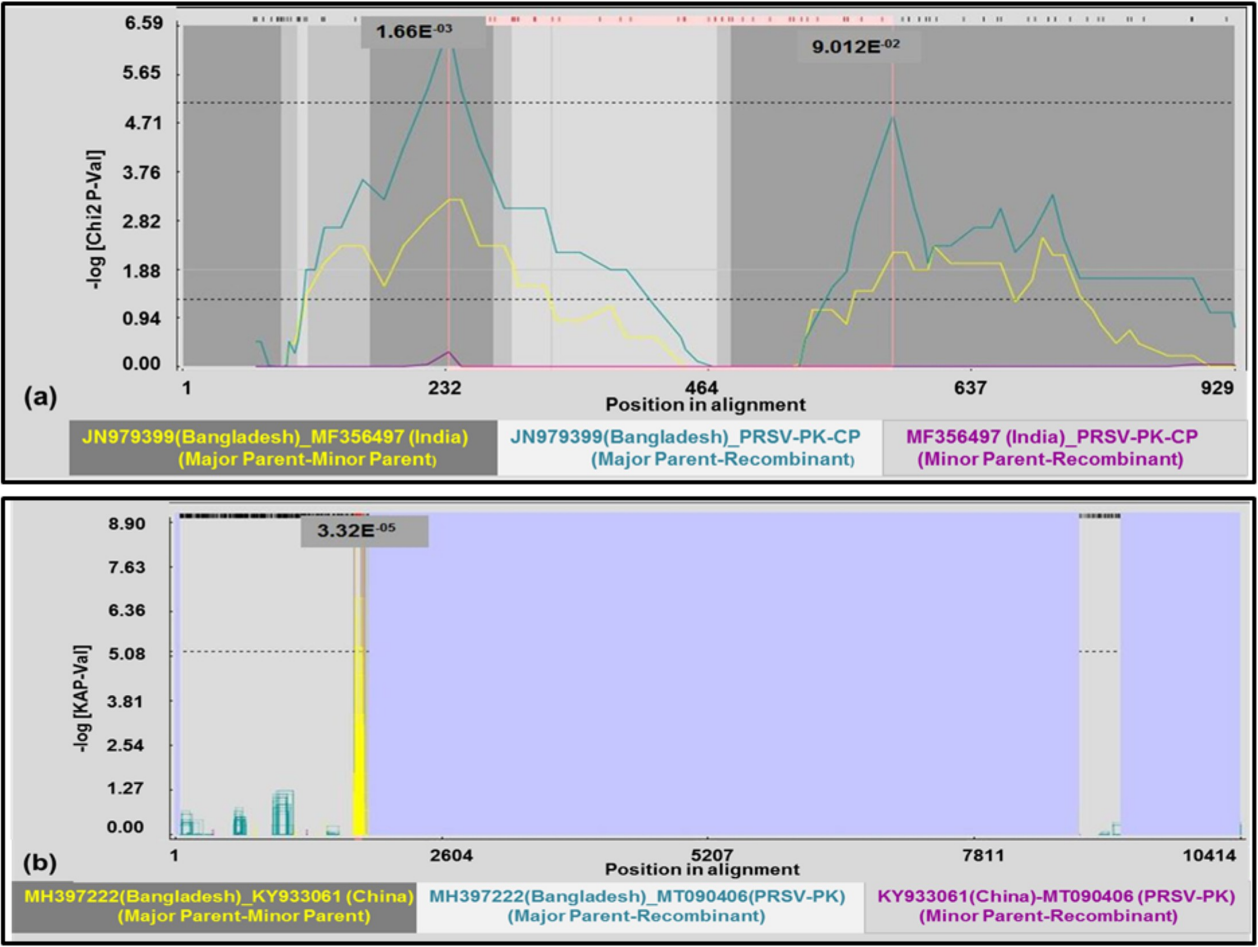
Recombination detection in PRSV-PK-CP gene and full-length genomic sequence. a) Maxchi plot built in RDP indicting the potential breakpoints in the recombinant PK-CP gene b) GeneConversion plot built in RDP indicating potential breakpoints in the full length PRSV-PK genome.

Recombination events in the CP domain of MT090406-PRSV-PK strain and other 34 geographically distinct PRSV isolates were analyzed by using GENECONV, BOOTSCAN, MaxChi, CHIMAERA, SiSCAN, 3SEQ and Lard within RDP4 package software. The breakpoints at gene positions (235–627) were observed and PK-PRSV-CP isolates were identified to be a potential recombinant by 4 of 7 methods implemented in RDP4. The major parent detected by the event is JN979399 (Bangladesh). The recombination event along with the breakpoint position and P-values is mentioned in the (Table 3) where recombinant analysis was positive with Chimaera (C), 3Seq (3S), MaxChi (M) and Lard (L), whereas; RDP (R), Gene Conversion (G), Bootscan (B) and Siscan (S) revealed no evidence of recombination. The plot built in RDP indicting the potential breakpoints in the recombinant PK-CP gene by using Maxchi (M) method is shown in the Fig 4(A).

**Table 3:** Recombination detection in CP gene of atypical PRSV-P Isolate PK (MT090406).

Event No	Recombinant	Major Parent	Minor Parent	Break point	RDP (R)	*Gene Conversion* (G)	*BootScan* (B)	*MaxChi* (M)	*Chimaera* (C)	*Siscan* (S)	*3Seq* (3S)	Lard (L)
1	MT090406	JN979399	Unknown	235–627	NS	NS	NS	1.66E^-03^	1.80E^-03^	NS	1.56E^-02^	5.00E^-04^
	PRSV-PK	Bangladesh										

The break point listed here refer to their position in the alignment; RDP(R), GENECONV(G), BOOTSCAN(B), MaxChi(M), CHIMAERA(C), SiSCAN(S), 3SEQ(3S), and Lard (L).

Recombination events in full-length genome of PRSV-P (PK) along with other reported PRSV full- length genome sequences from Hawaii, Taiwan, France, USA, Colombia, India, China, Bangladesh, New-Guinea and Thailand were detected using GENECONV, BOOTSCAN, MaxChi, CHIMAERA, SiSCAN and 3SEQ within RDP4 package software. The target genome was found to be highly recombinant with a total of 61 recombination events detected in RDP and plausible breakpoints were also identified in the particular event. Results are highlighted for three selected events including 28, 34 and 57 detecting PRSV*-*PK isolate to be recombinant by the methods within RDP software. Bangladesh isolate (MH397222) was detected to be the potential parent in event number 28 detected by GeneConversion, Chimaera and 3 Seq. The potential breakpoint during this event was (1760–1829) thereby indicating the recombination towards the 5’ terminal region of genome respectively. The RDP event number 34 also detected PRSV–PK as recombinant with the major parent from MF074214 (China) with the breakpoint position of (52–329). Another event number 57 with PRSV–PK as a recombinant showed a major parent form China (HQ424465) with the breakpoint position of (9340–9740). The detail of the recombination events showing PRSV–PK to be recombinant along with the breakpoints position and the average P–value is mentioned (Table 4). The plot showing the PRSV-PK as recombinant along with the breakpoint distribution detected by at least one method (GeneConversion) is mentioned in Fig 4B.

**Table 4:** Recombination detection in full length atypical PRSV–P Isolate PK (MT090406).

Sr. No/Event No	Recombinant	Major parent	Minor Parent	Breakpoints	RDP (R)	Gene Conversion (G)	BootScan (B)	Maxchi (M)	Chimaera (C)	Siscan (S)	3Seq
**1(28)**	MT090406	MH397222	KY933061	1760–1829	NS	3.32E^-05^	NS	NS	4.77E^-02^	NS	**9.63E** ^**-06**^
	PRSV-PK	Bangladesh	Fujian China								
**2(34)**	MT090406	Unknown	MH404260	52–329	-4.71E^-05^	-NS	1.65E^-03^	-NS	NS	1.52E^-07^	**NS**
	PRSV-PK	MF074214	New Guinea								
		Hainan									
**3(57)**	MT090406	HQ424465	EF017707	9340–9740	2.32E^-03^	NS	5.17E^-04^	NS	NS	1.32E-^14^	**NS**
	PRSV-PK										

The break points listed refer to their positions in the alignment; RDP(R), GENECONV(G), BOOTSCAN(B), MaxChi(M), CHIMAERA(C), SiSCAN(S), 3SEQ(3).

## Discussion

**Fig 5:**
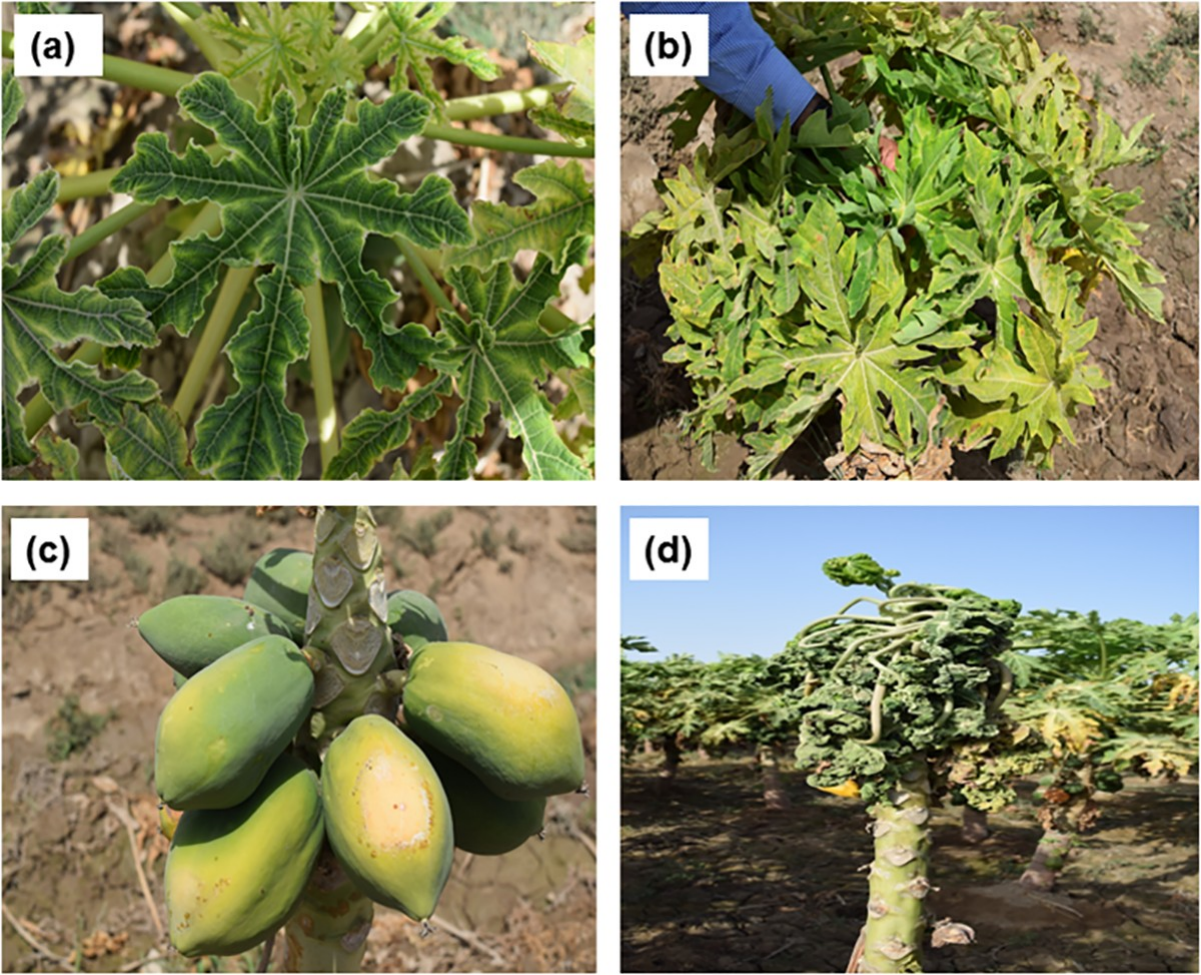
Symptoms induced by PRSV-PK under field conditions. a) Discoloration on the individual leaves b) Bunch of infected leaves showing yellowing c) stunted fruit with the appearance of ringspot d) whole papaya tree destruction.

Since the introduction of papaya in the cropping system, incidence and damage caused by the aphid transmitted *Papaya ringspot virus* (PRSV) has been increasing over time globally [44]. In the realm of pathogen-derived systemic resistance strategies for management of PRSD, transgenic antiviral papaya was effectively utilized to overcome widespread invading PRSV [45–47]. Nevertheless, reports of recent outbreaks in China has shown a concomitant decline in transgenic resistance because of occurrence of resistant viral strains in Hainan province, thus leading to loss of resistance and massive crop damage [48]. Since occurrence of the PRSV and subsequent infection of cultivated papaya in Pakistan, preliminary scientific investigations have shown emergence of an atypical PRSV in Pakistan on the basis of viral coat protein gene [15]. Earlier findings have also indicated that PRSV biotype W infecting cucurbits in Pakistan remains atypical in the pre-history of potyviruses on the basis of its divergent CP gene [14]. Our follow-up studies, on PRSV infection in papaya grown at a large scale in Pakistan have shown the overall estimated loss of 80–100% in all major orchards particularly in Sindh province (Fig 5). Here, we present the first annotated genome sequence of *Papaya ringspot virus* biotype P from Pakistan (PRSV–P PK), of 10,320 nucleotides deposited in the GenBank with accession number MT090406. This sequence corresponds to the viral strain found to be most prevalent (88%) among all isolates of PRSV from Pakistan. The aggressive symptomatic appearance and a higher rate of infection in papaya crop plants with this atypical PRSV strain, yield losses up to 100%, have been recorded in surveys and analyses. The occurrence and disease tendency required further genetic variability and genome recombination analysis of the virus to estimate its provenance in a global perspective. It has already been speculated that the emergence of PRSV-P in earlier identification is related to its counterpart PRSV-W recorded five years before in the country [15].

The abrupt spread of the disease caused by PRSV was first surveyed in 2011 from Malir district Karachi and later surveys confirmed the disease in Islamabad (NARC field papaya). The damage caused by PRSV isolate PK under field conditions has reached up to 100% [49]. The field survey and analysis of symptoms reveal excessive damage of papaya, symptoms observed on leaves (Fig 5A and 5B), stunted fruits (Fig 5C) and whole tree destruction (Fig 5D) conclusively shown in Fig 5. Infectivity assay is the key for the evaluation of pathogenicity assessment and fulfillment of Koch’s postulates [50]. Similarly, the infectivity assays were performed on the available PRSV hosts including *C*. *quinoa*, *C*. *metuliferous* and *C*. *papaya* assuring the variable nature of virus infections on multiple hosts. The virus induced typical local lesion on indicator host, *C*. *quinoa* and systemic infection followed be vein clearing, mild to severe mosaic and leaf shoe string on papaya and *C*. *metuliferous*.

The mutation-prone RNA genome of PRSV has evolved over time showing remarkable evolutionary behavior leading to genetic diversity [48]. Efforts to find the origin and distribution of PRSV targeted CP gene (the CP gene position is 9258–10117 in the complete genome) sequence revealed that isolates from Mexico corroborate maximum genetic similarity with USA and Australian isolates and clear separation from Asian PRSV isolates including those isolated from the Indian continent [11, 51]. The isolates analyzed on the basis of CP gene from India showed a divergence of 0–11% which do not differ remarkably from reported variation with Bangladesh isolates (9–14%), the rest of the Asian PRSV population (4–14%), and with Australia/America (5–11%) (Fig 2A). In homology studies based on CP gene, PRSV–PK showed the highest relatedness with previous PRSV isolates from Pakistan whereas it is distantly related to PRSV isolates from Bangladesh and India (Meghalaya and West Bengal) (Fig 2A), It is worthy to mention that PRSV isolates from Bangladesh and India (Meghalaya) have occurred after the establishment of atypical PRSV in Pakistan. As the PRSV–PK strains emerged as distinct subpopulation of the virus since its inception, it later diverged from the other Indian isolates from Pune [52], and Delhi [53] as well. The possibility behind the divergent nature of the Pakistani PRSV strain could be due to the occurrence of recombination events in the amino-terminal of CP region where some sequence variation in other variant of this virus has been reported [54]. However structural and functional integrity of PRSV has maintained [11]. Our study showed the full-length CP gene of PRSV-PK comprises of only 849 nucleotides with the deletions at the 5’ terminal of CP (nt position 9318) and at position (9346) with an overall 6% variation in the 5’ terminal region corresponding to EK aa repeats deletion. This further points to the occurrence of substitutions at amino acid positions 122, 157 and 244 in CP protein causing differentiation of the PRSV-PK in comparison to CP protein worldwide.

**Fig 6:**
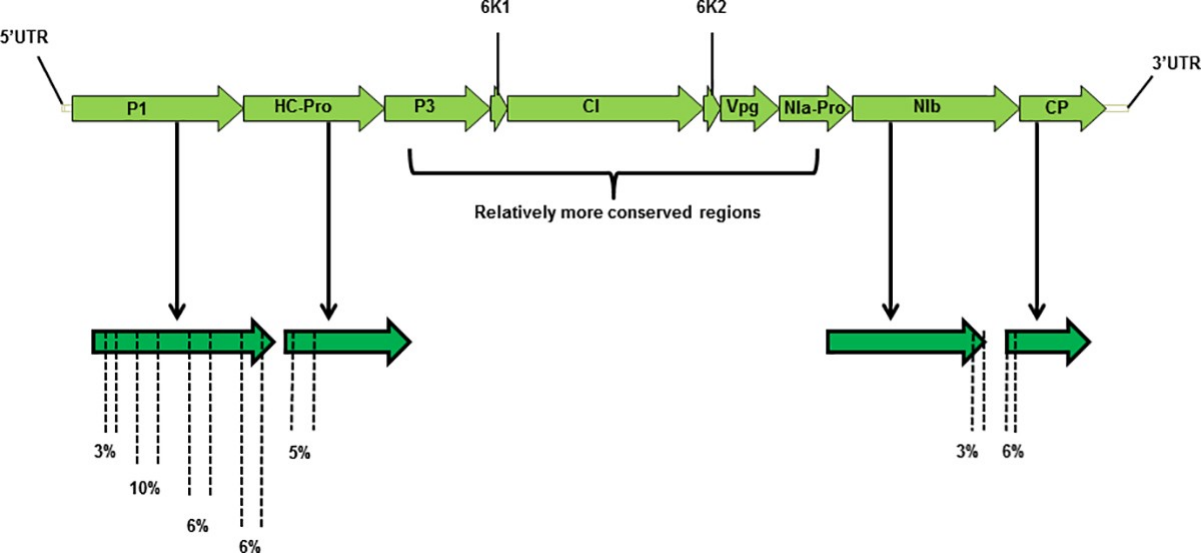
Approximate genetic variation in the individual gene fragments of PRSV-PK.

The P3, CI, Vpg, NIa-Pro appeared relatively conserved at the nucleotide and amino acid sequence level in comparison to other isolates of PRSV globally (Fig 6). Therefore, variable genome components P1 and HC-Pro of PRSV-PK were selected for individual gene analysis. The regions of variation have been demonstrated in Fig 6, showing that PRSV-PK isolate possesses nucleotide and amino acid variation in P1 and HC-Pro genes as well; eventually, corresponding to divergence of this particular virus from other isolates of regional or distinct geographical locations. The length of P1 gene appears to be 1647 nucleotides with a high level of evenly distributed genetic variation 3% towards 5’, up to 10% in the middle, and 6% towards the 3’ termini. Variable nature of P1 has also been reported in earlier studies [10, 24, 55]. The HC-Pro gene length is 1370 nucleotides with approximately 5% genetic variation at the immediate 5’ terminal region.

Despite high genome variation throughout P1 and HC-Pro genes of PRSV-PK isolate both clustered with an isolate from Bangladesh (MH397222) in phylogenetic analysis. The high variation in P1 gene of PRSV-PK as mentioned in (Fig 6) is the possible reason for its relatively low affinity towards PRSV Bangladesh isolate BD2 (homology 85%). Similarly, HC-Pro appears to have high genetic divergence from other isolates worldwide with several interlinking clusters that appeared in phylogeographic analysis (Fig 2C).

**Fig 7:**
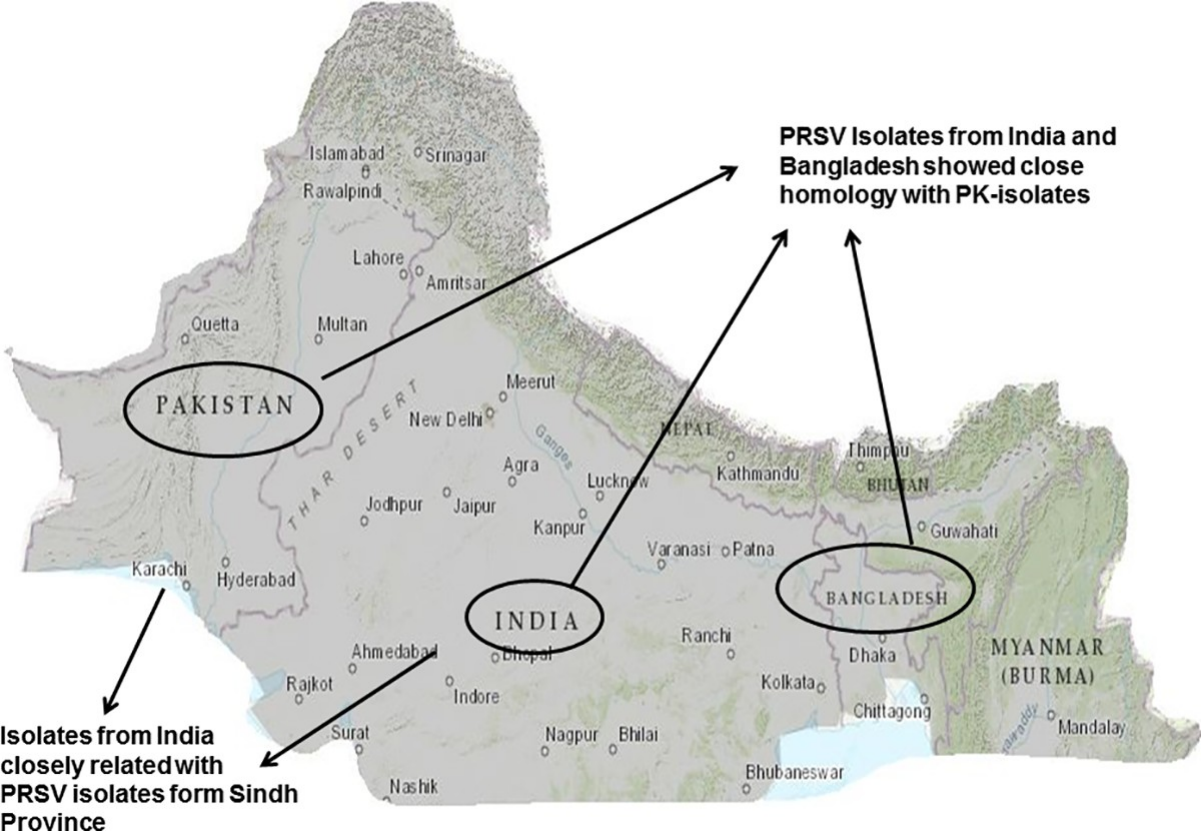
Map showing the isolates of PRSV–P of Pakistan compared on the basis of coat protein sequence showed close homology to Bangladesh isolates and some of the Indian isolates.

Initially the 3’UTR region of PRSV was considered relatively conserved because of its least vulnerability towards recombination [10, 24]. However, in our findings, 3’UTR phylogenetic analysis distinctly made three clusters (Fig 2D). The phylogenetic analysis based on PRSV complete genome sequences revealed 89.8% homology of PRSV–P isolate PK (MT090406) with PRSV–Bangladesh (MH397222). Thus, the closest relative appeared recently annotated complete genome sequence of PRSV from Bangladesh (MH397222) [24]. Another closely related isolate to PRSV-PK in the same clade is a recently sequenced isolate from West Bengal-India (LC482263) [25] with (89.2%) homology. The PRSV isolates from northern and western regions of India form a genetically distinct cluster (Fig 3). This distinct clustering complies with the clustering of PRSV isolates of western Indians with those of Americans [56] Similar Indian and American isolates clustering appeared in the comparative neighbor-joining trees based on full-length PRSV genomes at amino acid level concluding that American PRSV strains have originated from India [10]. Thereby, strengthening the postulate of the emergence of the distinctive virus in the Indian subcontinent, it holds the oldest PRSV population and that the actual origin of this virus had already been scientifically speculated in the South Asia [50] where we reinforce that changes in the length of CP gene could be a potential reason of genetic variation [21, 54]. Nevertheless, the biogeographical analysis of the complete genome of PRSV-PK fairly prospected that this atypical PRSV strain emerged and evolved in Indian Peninsula (Fig 7). The dispersal is spreading in Asian regions including India, Pakistan, and Bangladesh and may be taking route to the rest of other Asian countries. In past, major dispersal events of PRSV have provoked devastation to papaya in the US mainland to Hawaii, and recently the breakup of transgenic resistance in China. However, there is no known present evidence or correlation that this atypical Asian strain pre-ambling replacement of PRSV Americas-Australia cluster. Furthermore, the PRSV-PK isolate that groups together with designated Indian and Bangladeshi isolates occurred so far from a separate dispersal event; not the same as apparent with the rest of the Asian regions including Thailand, China and Taiwan. In another report of only CP based comparative analysis, Indian and Bangladesh isolates are separate from other Asian isolate’s cluster therefore, depicting that clustering of PRSV could not be directly linked with the geographical origin [21]. We also agree that the movement and dispersal events lead to the occurrence of single and mixed populations or subpopulations of the isolates.

The RNA viruses mark recombination and genetic differentiation which can lead to evolution as biodiversity [48]. The study on genetic differentiation and gene flow between PRSV populations from diverse localities is of critical importance keeping in view this newly emerged atypical PRSV cluster from India, Bangladesh and Pakistan. The gene flow and genetic differentiation between PRSV populations conducted using DnaSP provided a comparative analysis of DNA polymorphism suggested functional significance of evolutionary process in genomic regions of PRSV. Analysis of differentiation between phylogroups based on CP sequences revealed complete differentiation as indicated by the significant Kst*, Snn and high Fst values >0.3. The previous reports on genetic differentiation analysis between virus populations having Fst values above 0.25 designate high genetic differentiation and infrequent gene flow between the populations [48, 57]. Consequently, there exists high genetic differentiation between the virus population of Pakistan and the virus population from Bangladesh, India, Mexico, America and Australia. Similar results based on the highest Fst (0.54) value between South America and Japanese populations using *Potato virus X*, CP gene sequences suggest infrequent gene flow attributed to the long distances between these geographic regions [58]. In Genetic differentiation assessment between PRSV full length genomic populations with the exception of Pakistan vs Asian population, all the Kst* values range between 0.015–0.25 and a bit higher than “0” supported by P values >0.05 suggesting that populations are not well differentiated. Similar results were presented showing no genetic differentiation and non-significant Snn values calculated by pairwise comparison of *Zucchini yellow mosaic virus* sequences between populations of Asia vs. Europe and Oceania vs. Africa [57]. Moreover, the Fst values generated between populations based on full genome of PRSV-PK as mentioned in Table 2 indicates the lowest Fst value of 0.26884 between PK vs Bangladesh suggesting frequent gene flow between them. High Fst values between rest of the populations suggests infrequent gene flow as Fst >0.33 is its indicator [34]. Fst values associated with free, high and moderate gene flow have also been described in gene flow studies of *Potato virus Y*, where, higher Fst values >0.6 depicts less frequent gene flow and low genetic exchange between populations [59]. The highly variable nature of P1 gene as mentioned in Fig 6 suggested to conduct the genetic differentiation and gene flow assessment between PRSV populations from Pakistan with the populations from other geographical locations based on P1 gene. The P1 gene is under strong positive selection suggesting that it could be major determinant of host adaptation [55] postulating differentiation based on this gene of utmost importance. The genetic differentiation and gene flow assessment between PRSV population based on P1 gene showed non-significant P-values of Kst* and Snn indicated that populations are not well-differentiated (S3 Table). However highest Fst value (0.92427) was recorded between Pakistan and Colombia while the lowest (0.27652) between Pakistan and Bangladesh. Based on P1 population, gene flow estimated between the population of Pakistan and Bangladesh appears to be frequent (S3 Table) thus directly linked with the geographical isolation. HC-Pro, yet another gene of functional significance is reported to be a strong silencing suppressor in potyviruses [55] was also explored for genetic differentiation and gene flow assessment. Non-significant P-Values of Kst* and Snn were observed upon comparison of PRSV–Populations based on HC-Pro gene again depicting that populations are not well differentiated (S4 Table) However, the lowest Fst values (0.32770) corresponds to the PRSV-PK-HC-Pro vs Bangladesh-HC-Pro populations thereby suggesting that there exists some level of frequent gene flow between these populations. It can be concluded that higher Fst values and low genetic exchange depicts correlation between geographical isolation and gene flow. A Similar correlation has been discussed for *Zucchini yellow mosaic virus* [57]. Thus, geographic isolation based on infrequent gene flow particularly between regions (PK vs America, PK vs Colombia, PK vs Mexico, PK vs other Asian regions revealed specific points of PRSV genome differentiation to give rise to PRSV-PK. Similarly, the infrequent gene flow based on *Sugarcane yellow leaf virus* (SCYLV) population from within Africa and between Asia and America, contributed to shape the genetic structure of SCYLV [60]. Yet another relatable interlinking of geographical isolation and genome architecture in plant virus with positive sense RNA genome has been reported recently for gene flow assessment among the population of *Maize yellow mosaic virus* (MaYMV) [61].The infrequent gene flow and high genetic differentiation between PRSV–PK populations based on CP gene also suggests that PRSV-PK may has evolved independently. However, large number of fully sequenced genomes from Bangladesh, India and Pakistan would further strengthen the reported evidence of genetic differentiation and gene flow assessment of this inter-Asian geographical clade (Bangladesh, India and Pakistan).

Other important factors that strongly influence the virus evolution include host adaptability and geo-climatic conditions. The climatic conditions of particular geographical locations play a part in shaping the phenotypic and genotypic properties of flora present in that location. Thereby changing climatic conditions will lead to the change in both phenotype and genotype of the existing host [62, 63]. Similarly, the host plants from different geographical locations possessing diverse genetic makeup in turn will contribute in shaping the invading virus population [64]. Hence the diverse and atypical nature of PRSV–PK is attributed to its adaptions to diverse ecological location and of course the host. In Pakistan, the local papaya varieties belong to Keto Bunder area and were cultivated largely long ago in the Coastal area of Sindh Province. Later hybrid papaya varieties were also planted and cultivation was extended from the Southern to the central part of the country. Therefore, papaya grown in Pakistan is not relevant to the papaya cultivated in India and Bangladesh which led to the emergence of unique PRSV. Even so, the host adaptability of PRSV population from different geographical locations regions is the major factor influencing plant virus evolution. The analysis of the diverse nature of three functional protein-coding regions, HC-Pro, NIa-Pro and CP of PRSV isolates from different geo-climatic regions and hosts also suggest that the PRSV population diversity is linked with the geo-climatic conditions as well as the host. However, it was also observed that different geo-climatic conditions affect the PRSV evolution with different levels of variations [23].

Recombination analysis of aligned CP sequences of 35 isolates including the Pakistan PRSV-CP was performed using RDP4 v 4.97. In our study single break point position was detected by 4 methods including Maxchi, Chimaera, 3Seq and Lard, pointing CP gene of PRSV-PK as potential recombinant with the break points at positions (235–627). Putative breakpoints positions at 208 and 605 in the CP gene of Colombian PRSV isolate was reported earlier [65]. The primary scans used in RDP indicating PRSV-PK CP gene as a recombinant region were also supported by the automated secondary scan implemented in Lard. The recombination probability derived through RDP in our study presents PRSV from Bangladesh as parent entity for PRSV-PK, while PRSV from India has been discussed as a minor parent entity of PRSV for Bangladesh [24]. The comparative recombination analysis between whole genome sequences clearly revealed that PRSV-PK representative isolate is closest with the recently reported Bangladesh isolate (MH397222) Furthermore, the recombination analysis of aligned PK-PRSV whole genome along with other PRSV isolates identified a total of 61 recombination events. The recombination sites were detected in the full-length PK-PRSV genomic sequence. The potential breakpoints detected in multiple events were 1760–1829, 52–329 and 9340–9740. The highest recombination was detected towards the 5’ region of the PRSV genome particularly including the first 1020 nt. Further, recombination hot spots were also detected at the NIb-CP junction of PRSV-PK as mentioned in event No 37 (Table 4). The potential regions prone to recombination are 5’UTR and P1, thus playing a major role in PRSV genome dynamics as this information strengthens some preliminary findings [56], followed by HC-Pro gene [66] and consistent with other potyviruses [67] as well. We conclude that PRSV from Bangladesh (MH397222) acts as a major parent in this recent outbreak of recombination (Table 4). KY933061 (China) reported to be a minor parent of PRSV-PK in one of the detected events. The same isolate (KY933061) from China appeared to be potential parent of Bangladesh isolate in previous study [24]. The recombination rate was higher in Asian, particularly in Pakistan, Indian, China and Bangladesh isolates. Similar findings of higher recombination rates in Asian specifically Chinese and Indian isolates were put forward in a recent report [25]. As our recombination analysis comprised of all the recently sequenced genomes thereby it is noteworthy to mention the detected recombination in lately sequenced severe PRSV-P isolate from Ecuador (South America) MH974110 [68]. However, the Table 4 states only the recombination events of PRSV-PK. The detected breakpoints in the PK-CP gene, estimated by Maxchi method within RDP4 is mentioned in the Fig 4(A) whereas the detected breakpoints in full length genome detected by GeneConversion method within RDP4 package is mentioned in Fig 4(B). The circle of PRSV recombination in globe is yet occurring dramatically. Earlier global prospects on spread of PRSV suggested its origin in India about 2250 years ago and its spread to Thailand and China about 600 years ago, eventually reaching to America about 200 years ago. At the same time keeping in the fact of PRSV-P origin from W biotype it can also be assumed that initially the virus found a slot in hosts of *Cucurbitaceae* family of particularly American origin thus leading to its introduction to America probably as Biotype-P or biotype-W via already widespread cultivated cucurbits species in South and Central America [69]. Since all species of the *Cucurbitaceae* family are not natural hosts of the PRSV-P [70] therefore it can also be assumed that type P evolved from type W and showed diversification in host adaptation most likely under influence of different geoclimatic conditions. Also, there is established link between transformation of invading virus population with genetic makeup of host plant [64]. Furthermore, characterization of full genome of a greater number of PRSV–W biotype is required to elucidate and validate the actual origin of this virus as well as its evolutionary linkages. The genetic differentiation, gene flow and recombination analysis highlighted the constant divergent behavior as well as atypical nature of the presented virus, the hotspots of virus origin, interlinking of the virus origin and epidemiological behavior with other isolates of variant geographical locations.

Papaya crop originated in Southern Mexico and Costa Rica, remained cultivated in the USA, India, Brazil, Mexico, Nigeria, Jamaica, Indonesia, China; Taiwan, Peru, Thailand, and Philippines to a status of economy boost up crop [71]. The emergence and spread of atypical PRSV have essentially been pointed out to provide updated knowledge of the recombination and genetic variation required for defining management and prevention strategy before it escalates to pose havoc on regional to global papaya production, owing to papaya as the economy boost up crop in many cultivating countries.

USGS EROS (Earth Resources Observatory and Science (EROS) Center) (public domain): http://eros.usgs.gov/#

## Conclusions

The investigation of Pakistan’s PRSV isolate shows its atypical “Unique” nature. The phylogenetics of the identified strain demonstrates how this strain is divergent from other geographically distinct strains. This trend of differentiation of PRSV populations also holds true for the genetic differentiation analysis done with other rest of populations from diverse geographical locations. The gene flow between PRSV populations from Pakistan and the virus populations from other localities as well as the predicted recombination in the identified strain also supplement the unique nature of our strain. Through the emergence of the atypical PRSV-PK isolate, this study contributes to understand the evolutionary divergence of this strain with respect to other strains from all over the world. This analysis is useful in understanding the how the evolutionary status of this virus has implications for the management strategies. This evolutionary and epidemiological study would not only facilitate to devise effective management strategy but will also help in mitigating the damage caused by PRSV-PK to papaya a plant with the potential to become a cash crop in Pakistan.

## Supporting information

S1 Table Primers used for schematic amplification of PRSV-P Isolate PK genome. (DOCX)

S2 Table The sources of CP, P1, HC-Pro, 3’UTR and whole genome sequences of PRSV isolate of Pakistan and other countries used in the study. (DOCX)

S3 Table Statistical tests for genetic differentiation and gene flow between *Papaya ringspot virus* populations from Pakistan with the populations from India, Other Asian Countries (Thailand, Taiwan, China), America, Bangladesh and Colombia based on P1 gene nucleotide sequences. (DOCX)

S4 Table Statistical tests for genetic differentiation and gene flow between *Papaya ringspot virus* populations from Pakistan with the populations from India, Other Asian Countries (Thailand, Taiwan, China), America, Bangladesh and Colombia based on HC-Pro gene nucleotide sequences. (DOCX)

S1 Fig Neighbor-Joining tree representing phylogenetic relationships of *Papaya ringspot virus*-P strain PK (PRSV–PK). (a) Coat Protein (CP) sequence (b) Protease P1 sequence (c) Helper component HC-Pro Sequence (d) 3’UTR sequence from other related sequences selected via BLAST search. Corresponding CP, P1, HC-Pro and 3’UTR sequences of *Sugarcane mosaic virus* (SCMV) (accession no. MN795295) used as outgroup. Upper and lower branch points show bootstrap values (1,000 replicates) supporting a particular phylogenetic group. The scale bar represents nucleotide substitutions per site. All nucleotide sequences are retrieved according to the isolate name and the GenBank accession number.
(TIFF)

S2 Fig Region-specific Neighbor-Joining tree representing the phylogenetic relationship between complete genome sequences of PRSV-P isolate of Pakistan and PRSV complete genome sequence from rest of the world. *Sugarcane mosaic virus* (SCMV) (accession no. MN795295) used as an outgroup. The tree was constructed using ClustalX2 and MegaX Program. The scale bar represents nucleotide substitution per site, the bootstrap value of 1,000.
(TIFF)

S1 File Sequence information (PRSV-Pakistan strain, Accession No, MT090406). (DOCX)
