# Supplementary material for: Genetic variability and evolutionary dynamics of atypical Papaya ringspot virus infecting Papaya
Source: PLoS One. 2021 Oct 12;16(10):e0258298. doi: 10.1371/journal.pone.0258298 (PMC8509892; doi:10.1371/journal.pone.0258298)
Supplement: S1 Table — (DOCX) [file pone.0258298.s001.docx]

**S1 Table. Primers used for schematic amplification of PRSV-P Isolate**

**PK genome**

| **Primers** | **Primer sequence (5'-3')** |
| --- | --- |
| 8701 Fwd | TCTCT GTATC GCCAT TCACC CGGAT C |
| HC-Pro India Fwd | GGGGAGAGTTATTGACGAAGTTG |
| HC-Pro India Rev | GTAACACAGCTCCAAACATGAACGT |
| PRSV CP Rev | CGACTGATGGTAGACGTGCTTG |
| PRSV 5110 Fwd | GCCACAAATATCATCGAGAATGG |
| PRSV 3870 Rev | CCTTCTGTATCTCCACTTCTGCC |
| HC-Pro Rev 2 | GACCGTTTATTTCTAAAGCTCTCAAC |
| HC-Pro PK Fwd | GGTGAGAGACAATATCATGCCA |
| PRSV 5' UTR Fwd | AAATAAAACATCTCAACACA |
| 3'UTR Rev | CTCTC ATTCT AAGAG GCTCG AATAG C |
