# Supplementary material for: Genetic variability and evolutionary dynamics of atypical Papaya ringspot virus infecting Papaya
Source: PLoS One. 2021 Oct 12;16(10):e0258298. doi: 10.1371/journal.pone.0258298 (PMC8509892; doi:10.1371/journal.pone.0258298)
Supplement: S2 Table — (DOCX) [file pone.0258298.s002.docx]

**S2 Table. The sources of CP, P1, HC-Pro, 3’UTR and whole genome sequences of PRSV isolate of Pakistan and other countries used in the study**

| **Accession number** | **Origin** | **Pathotypes** | **Gene segment/genome** | | | | |
| --- | --- | --- | --- | --- | --- | --- | --- |
|  |  |  | **CP Gene** | **P1 Gene** | **HC-Pro Gene** | **3'UTR** | **Complete PRSV genomes** |
| MT090406 | Pakistan | Type P | 849bp | 1647bp | 1371bp | 209bp | 10320 |
| X97251 | Taiwan | Type P | 855bp | - | - | 209bp | 10326 |
| JX448372 | Taipie | Type P | - | - | - | 206bp | 10326 |
| EU882728 | Taiwan | Type P | - | - | - | 206bp | 10320 |
| X67673 | Hawaii | Type P | - | 1641bp | 1371bp | 206bp | 10326 |
| S46722 | Hawaii | Type P | 864bp | - | 1371bp | 206bp | 10326 |
| EU126128 | Hawaii | Type P | NA | - | - | 206bp | 10320 |
| EF017707 | India | Type P | 857bp | - | 1371bp | 206bp | 10317 |
| MH311882 | Pune | Type P | NA | - | 1371bp | 207bp | 10326 |
| MF405299 | Pune (Aundh) | Type P | NA | - | - | - | 10341 |
| MF405298 | Pune (Aundh) | Type P | NA | - | - | - | 10341 |
| MF405297 | Pune  (Aundh) | Type P | NA | - | - | - | 10341 |
| MF405296 | Pune (Aundh) | Type P | NA | 1641bp | - | - | 10341 |
| MF356497 | Meghalaya | Type P | 861bp | 1641bp | 1371bp | 223bp | 10343 |
| MF074214 | Hainan (China) | Type P | 924 | - | 1371bp | 204bp | 10327 |
| HQ424465 | Hainan | Type P | NA | - | 1371bp | 206bp | 10332 |
| KF791028 | Hainan | Type P | NA | - | - | 208bp | 10326 |
| EF183499 | Hainan | Type P | NA | - | - | - | 10323 |
| KY271954 | PTX | Type P | 914 | 1641bp | 1370bp | 210bp | 10320 |
| KT275938 | Colombia | Type P | 861bp | 1641bp | 1370bp | 206bp | 10326 |
| KT275937 | Colombia | Type P | 860bp | 1641bp | - | 206bp | 10326 |
| KC345609 | France | Type P | 860bp | 1641bp | - | 227bp | 10343 |
| AY162218 | Thailand | Type P | NA | 1641bp | 1371bp | 206bp | 10323 |
| AF196839 | USA | Type P | 858bp | - | - | NA | NA |
| AB044342 | Malaysia | Type P | 864bp | - | - | NA | NA |
| AF063220 | India | Type P | 855bp | - | - | 206bp | NA |
| DQ192587 | India (Pune) | Type P | 855bp | - | - | NA | NA |
| KC149502 | India | Type P | 858bp | - | - | NA | NA |
| JQ394695 | Bangladesh | Type P | 849bp | - | - | NA | NA |
| AJ875104 | Veitnam | Type P | 819bp | - | - | NA | NA |
| AF196838 | Florida | Type P | 858bp | - | - | NA | NA |
| AF344647 | Brazil | Type P | 864bp | - | - | NA | NA |
| AF506902 | Phillipines | Type P | 834bp | - | - | NA | NA |
| AJ012649 | Mexico | Type P | 858bp | - | - | NA | NA |
| AJ012650 | Mexico | Type P | 858bp | - | - | NA | NA |
| AY01072 | Thailand | Type P | 861bp | - | - | NA | NA |
| D50591 | Japan | Type P | 861bp | - | - | 206bp | √ |
| DQ419573 | Hainan | Type P | 855bp | - | - | 239bp | √ |
| JN132471 | Oklahoma | Type W | 852bp | - | - | NA | NA |
| KC768854 | Cuba | Type W | 834bp | - | - | NA | NA |
| JN979406 | Bangladesh | Type P | 852bp | - | - | NA | NA |
| JN979399 | Bangladesh | Type P | 849bp | - | - | NA | NA |
| MG383641 | India | Type P | - | - | 1371bp | NA | NA |
| MF481203 | India (Banglore) | Type P | - | - | 1350bp | NA | NA |
| KY448319 | India (Delhi) | Type P | - | - | 1370bp | NA | NA |
| KY448321 | India Delhi | Type P | - | - | 1370bp | NA | NA |
| KY448320 | India Delhi | Type P | - | - | 1370bp | NA | NA |
| KY448322 | India Goa | Type P | - | - | 1370bp | NA | NA |
| KU359197 | India Delhi | Type W | - | - | 1370bp | NA | NA |
| KU196789 | India Delhi | Type P | - | - | 1370bp | NA | NA |
| KU196790 | India Delhi | Type P | - | - | 1370bp | NA | NA |
| KU196791 | India UP | Type P | - | - | 1370bp | NA | NA |
| HQ328803 | Colombia | Type P | - | - | 1371bp | NA | NA |
| EU556733 | India Delhi | Type W | - | - | 1371bp | NA | NA |
| U14740 | Australia | Type-P | 842bp | - | - | - | - |
| U14744 | Australia | Type-W | 842bp | - | - | - | - |
| JX025001 | PK | Type-P | CP | - | - | - | - |
| JX025000 | PK-TJ | Type-P | CP | - | - | - | - |
| JX025002 | PK-TJ | Type-P | CP | - | - | - | - |
| JX024999 | PK | Type-P | CP | - | - | - | - |
| JX661503 | PK-Belli | Type-P | CP | - | - | - | - |
| JX661506 | PK-Belli | Type-P | CP | - | - | - | - |
| JX661507 | PK | Type-P | CP | - | - | - | - |
| JX661504 | PK-Dar | Type-P | CP | - | - | - | - |
| JX661505 | PK | Type-P | CP | - | - | - | - |
| MH397222 | Bangladesh | Type-P | 850bp | 1647bp | 1370bp | 206bp | 10325 |
| MH444652 | Bangladesh | Type-P | 864bp | 1632bp | 1371bp | 206bp | 10300 |
| LC482263 | India | Type-P | 852bp | 1647bp | 1371bp | 222bp | 10343 |
| KP743981 | Hyderabad | Type-P | - | - | - | - | 10341 |
| MF405295 | India | Type-P | - | - | - | - | 10341 |
| JX448373 | Taiwan | Type-P | - | - | 1371bp | - | 10326 |
| JX448371 | Taiwan | Type-P | - | - | - | - | 10326 |
| MH404262 | New Guinea | Type-P | - | - | - | - | 10315 |
| MH404259 | New Guinea | Type-P | - | - | - | - | 10301 |
| MH404260 | New Guinea | Type-P | - | - | - | - | 10354 |
| MH404263 | New Guinea | Type-P | - | - | - | - | 10351 |
| KT895257 | China | Type-P | - | - | - | - | 10332 |
| MF085000 | Shandong China | Type-P | - | - | - | - | 10337 |
| KY933061 | Fujian China | Type-P | - | - | - | - | 10326 |
| MH974110 | Ecuador | Type-P | 864bp | 1641bp | 1371bp | 204bp | 10324 |
