# Supplementary material for: Genetic variability and evolutionary dynamics of atypical Papaya ringspot virus infecting Papaya
Source: PLoS One. 2021 Oct 12;16(10):e0258298. doi: 10.1371/journal.pone.0258298 (PMC8509892; doi:10.1371/journal.pone.0258298)
Supplement: S3 Table — (DOCX) [file pone.0258298.s003.docx]

**S3 Table. Statistical tests for genetic differentiation and gene flow between *Papaya ringspot virus* populations from Pakistan with the populations from India, Other Asian Countries (Thailand, Taiwan, China), America, Bangladesh and Colombia based on P1 gene nucleotide sequences.**

| **Populations** | **Kst*** | **P value** | **Snn** | **P value** | **Fst** |
| --- | --- | --- | --- | --- | --- |
| Pakistan vs Other Asian (Thailand, Taiwan, China) | -0.02781 | 0.3930^ns^ | 1.00000 | 0.1030^ns^ | 0.74802 |
| Pakistan vs Indian | -0.07819 | 1.0000^ns^ | 0.66667 | 0.1940^ns^ | 0.46883 |
| Pakistan vs American | 0.02750 | 0.4800^ns^ | 1.00000 | 0.0810^ns^ | 0.80487 |
| Pakistan vs Bangladesh | 1.00000 | 1.0000^ns^ | 0.50000 | 0.3380^ns^ | 0.27654 |
| Pakistan vs Colombia | 1.00000 | 1.0000^ns^ | 1.00000 | 0.3330^ns^ | 0.92427 |

ns, not significant; *0.01 < P < 0.05; **0.001 < P < 0.01; ***P < 0.001. *Kst*,* *Snn and Fst* were implemented in DnaSP 6. The deviation hypothesis from null population differentiation was tested by 1000 permutations of the raw data.
