# Supplementary material for: Genetic variability and evolutionary dynamics of atypical Papaya ringspot virus infecting Papaya
Source: PLoS One. 2021 Oct 12;16(10):e0258298. doi: 10.1371/journal.pone.0258298 (PMC8509892; doi:10.1371/journal.pone.0258298)
Supplement: S4 Table — (DOCX) [file pone.0258298.s004.docx]

**S4 Table. Statistical tests for genetic differentiation and gene flow between *Papaya ringspot virus* populations from Pakistan with the populations from India, Other Asian Countries (Thailand, Taiwan, China), America, Bangladesh and Colombia based on HC-Pro gene nucleotide sequences.**

| **Populations** | **Kst*** | **P value** | **Snn** | **P value** | **Fst** |
| --- | --- | --- | --- | --- | --- |
| Pakistan vs Other Asian (Thailand, Taiwan, China) | -0.01904 | 0.4710^ns^ | 1.00000 | 0.0670^ns^ | 0.67450 |
| Pakistan vs Indian | 0.01546 | 0.2550^ns^ | 1.00000 | 0.0090^**^ | 0.59388 |
| Pakistan vs American | 0.11839 | 0.1150^ns^ | 1.00000 | 0.0660^ns^ | 0.79116 |
| Pakistan vs Bangladesh | 1.00000 | 1.0000^ns^ | 0.75000 | 0.3160^ns^ | 0.32770 |
| Pakistan vs Colombia | 1.00000 | 1.0000^ns^ | 1.00000 | 0.3390^ns^ | 0.73479 |

ns, not significant; *0.01 < P < 0.05; **0.001 < P < 0.01; ***P < 0.001. *Kst*,* *Snn and Fst* were implemented in DnaSP 6. The deviation hypothesis from null population differentiation was tested by 1000 permutations of the raw data.
