# Supplementary figures and images for: Genetic variability and evolutionary dynamics of atypical Papaya ringspot virus infecting Papaya
Source: PLoS One. 2021 Oct 12;16(10):e0258298. doi: 10.1371/journal.pone.0258298 (PMC8509892; doi:10.1371/journal.pone.0258298)

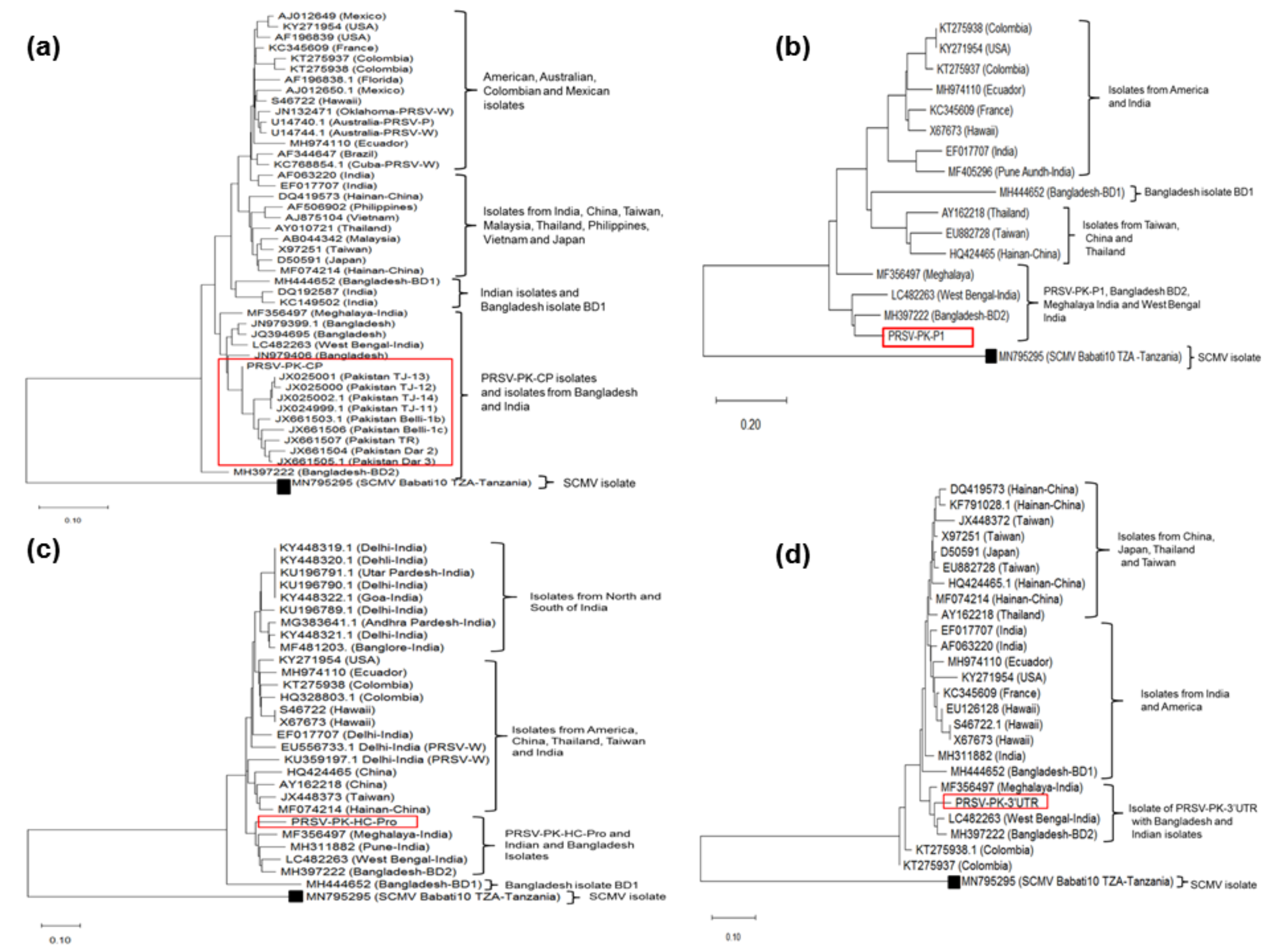

Supplement: S1 Fig — (a) Coat Protein (CP) sequence (b) Protease P1 sequence (c) Helper component HC-Pro Sequence (d) 3’UTR sequence from other related sequences selected via BLAST search. Corresponding CP, P1, HC-Pro and 3’UTR sequences of Sugarcane mosaic virus (SCMV) (accession no. MN795295) used as outgroup. Upper and lower branch points show bootstrap values (1,000 replicates) supporting a particular phylogenetic group. The scale bar represents nucleotide substitutions per site. All nucleotide sequences are retrieved according to the isolate name and the GenBank accession number. (TIFF) [file pone.0258298.s005.tiff]

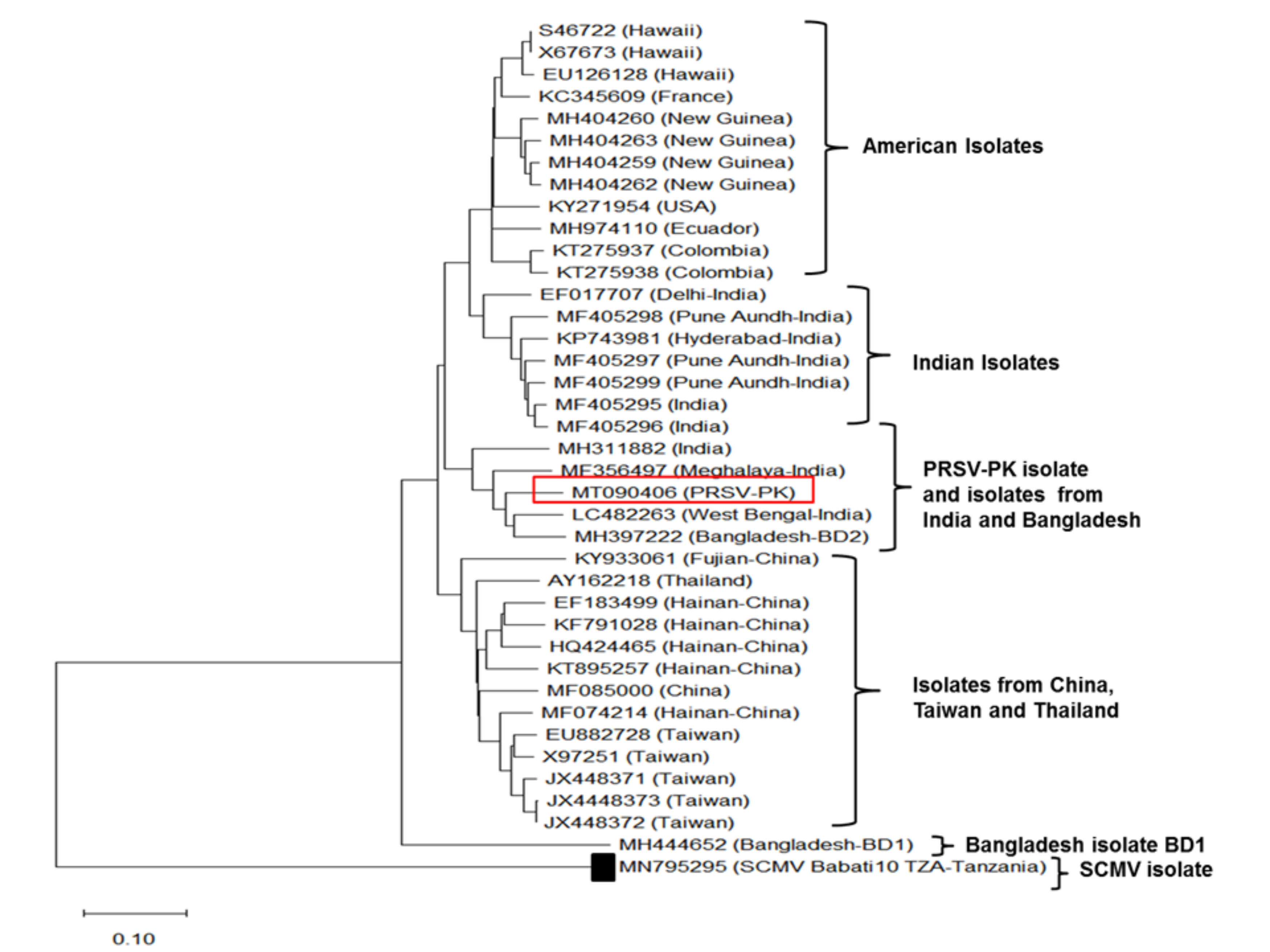

Supplement: S2 Fig — Sugarcane mosaic virus (SCMV) (accession no. MN795295) used as an outgroup. The tree was constructed using ClustalX2 and MegaX Program. The scale bar represents nucleotide substitution per site, the bootstrap value of 1,000. (TIFF) [file pone.0258298.s006.tiff]
