# Supplementary material for: Genetic variability and evolutionary dynamics of atypical Papaya ringspot virus infecting Papaya
Source: PLoS One. 2021 Oct 12;16(10):e0258298. doi: 10.1371/journal.pone.0258298 (PMC8509892; doi:10.1371/journal.pone.0258298)
Supplement: S1 File — (DOCX) [file pone.0258298.s007.docx]

>MT090406 (PRSV-Pak)

aaataaaacatctcaacacaacacaatcgaaaacaatcaaacaaatttcaactttgttcactatcgaatc

ttacaacaagcaatcatgtcttctctgtacacgctacgacctgctgctcaatacgataggagactagaaa

caaagaaaggttccagctggatcgagcgtaaactcgaatggaaaggtgaaagagggaacactcactattg

tagtgagtttgacataagcaagggtgccaagattctgcaattgattcagatcggcaatgctgaagttgga

agaacttttctggaaggtaacaagtttgttcgtgccaacatctttgaaatcgtcagaaagacgatggttg

gccacctggggtatgattttgagtgcgagctatggttctgtcacacttgcgacagaacatctgcgaagta

ctttaagaaatgtgagtgtggtgagaattattactattctgaaagaaatctaatgaaaacattgaataat

ctcacatatcaattcgaccttacgccatccgagattgatgcaatcgatctcgactacttagctgaagctg

tcgattacgctgaacaaagcgtgaaaaagacggcaatggttgaagatgcacccaatcctgtacaagaagt

agtgagcaacaaagtgtccactgtagttgatccaattataccaaatgcggtgccagccacgaataaagtg

gagcaagcgtggacaatacagattggcgatatcactgtgccactcgtagcaattgaagagacgccagtcg

tcagtggaatcattggttcattgaaatcaaccggtttctcggttgaggaagcaactgaaaagctagtgga

aactgaaattccacagtccaaaattgaggaagcagtgcatctggcactagaagtcggaaacgagattgct

gagaaaagacctgacttgaaattaataccatactggagtgctagtttggagatgcgcaaaagaatcagga

aacacattaacgatgctcgcgaaatgactaggttggtcaaagagcgacaggataaagacaagcaaatctt

tgattctctagagagacagctcaatctgaaagccagaaggaaggaacaggtcatcgtctgtgatcaaaga

ggaactctaaagtggaaaaagcgacagcaacgcaaaatgagcaggaaagtcttgcaaggatgtgattctg

ttatcacaaaaattcacggtatctctgaatatgagcttcagcataccgatttttccactcctggcattaa

atgcgcaacatcgaggaaagtccaaaggaaaaagaagggaagtcagtccaagataattggtagcagaaaa

atcaattatgtcatgagaaatatgtgtgatgtaatcatagaaaagaatatccctgttgaattaataacaa

agcgatgcaagagaagaatcttccgtaaagccggtagaaactatgtgcaactgagacacatggatggtat

caatgctaaacgagatgtgagaagttcaccagaaatggagaagctattcacgcagttttgcaggtttctg

attaggaagcaaacagttaaagcaaaaatgttggcacatggttcaagtggcctaatttttaagccaaagt

ttggtgataaaataggtcgatatttcggtgattacttcatcgttcgtggacgctatgaagggaaactgtt

tgatgggagatccaagttatcaaatccagtgcgattcaagatggagcagtatagtgatgtggccgagaaa

ttttggctcggttttaacagggccttcttgcggcataggaagccaacggatcatgtatgtacatccgaca

tggatgttacaatgtgtggtgaagtagctgccttagcaacgataatcctgtttccgtgtcacaagataac

ttgtaacacttgcatgaaccgggtaaaggaaagagtcattgatgaagtcggtgaagatctaaattgtgaa

cttggacgtttacgtgaaactctttcgtcgtacggaggctcattcgggcatgtctcagcgttacttgatc

aattgaacagagttttgaatgcacgcaacacgaatgatggagcttttaaggaaatagcaaagaaaatcga

tgataagaaagaaagcccctggacccacatgacagccatcaataatacgctcattaaaggttcgttagca

actggctacgaatttgaaagagcgtctgataatcttcgagagattgtgagatggcatctcaaaagaacgg

aatcaataaaggctggcagtgttgagagctttagaaataaacggtctggaaaagctcattttaatctagc

tcttacatgtgataaccaattggacaggaatggtaacttcgtgtggggtgagagacaatatcatgccaaa

agattctttgctaactattttgagaaaattgatcacagtaagggttatgagtactacagtcagcgccaaa

atccaaatggtgtcaggaaaattgcaattggaaacttgatattctcaacaaatttggagaggtttcgaca

gcaaatggttgaacatcacattgatcaaggaccaatcacccgtgagtgtatcgcattgcgcaataacaat

tacgttcatgtgtgtagttgcgttaccttggatgatggaactccagcaacaagtgagttgaagactccaa

ccaagaatcacattgttcttggtaattctggtgatcctaagtatgttgacttaccgactcttgagtctga

atcaatgtacatagctaagaaaggatactgctacatgaacatctttttggcgatgctcataaacatacct

gagaatgaggcgaaggattttacgaagagagttcgtgatcttgtgggttcaaaacttggagaatggccaa

cgatgttagatgctgccacatgcgccaatcagttggttatttttcatcctgacgcagctaatgcggaatt

gccaagaattctggtggatcaccgacagaagacaatgcatgtcattgattcgttcggatcagtggattct

ggataccacatattaaaagcaaacacagtcaatcagctgattcaatttgccagagaaccacttgacagtg

agatgaaacactatatagttggcggagaatttgatccaacgactagttgcttgcaccagttgattcgtgt

catttacaagccttatgaactccgagatttgctcagaaatgaaccgcatttaattgtgattgcattgatg

tcaccaagcgtgcttttaactctgttcaatagcggtgcaattgaacatgcgttgaattattggatcaaaa

gggatcaagatgttgttgaagttatagtcttggtggagcaattgtgcaggaaagtaacgcttgctaggac

aatcatggagcaattcaacgaaattcgtcaaaatgcaagagacatacatgagctgatggatcaaaatagt

aaaccttggatttcatatgatcgttcattagagttattgagtgtgtatgcaaattcacatttaacagatg

aaggcctactcaagcaaggattttctacactggaccctagactacgtgaagctgttgaaaaaacctacgc

cgctcttttgcaggaagagtggcgtgctttaagtttgtttcaaaagttgcatttaagatactttgcgttc

aaatcacaatcacctttttccgagtatttaaagccaaaagggcgcgcagatttaaaaattgtgtacgact

tctcaccgaagtactgtgcacacgaggtcggaaaggcgctgctacagccaatcagagctggagctgaaat

aacatcgcaaattgttagtggctgcggaacattcattcggaaaagcgctgcaaaaggttgtgcttacatt

ttcaaggatctttttcagttcgtgcacgtagttttagttttaagtattctactgcaaatatttaggagcg

tgcaaggaatcgccatggagcatatacaattaaagcaagcaaaggcagaaatggaaagacagaaagattt

tgatcagttagaagctctatacaccgaactgtgtgtcaagagcggtgaacagccaacagcagacgaattt

cttgatttcgtgacagaacgtgaaccaaggctcaagaatcaagtctgcaacttgattcacataccagtga

tacaccaagcaaaatctgacaatgaaaagaaacttgagcaagtaatcgcattcatcacattaattttgat

gatgattgacgtggataagagtgattgtgtgtatagggtcttgaacaagtttaagggtgtgataaagtct

tgtgatacaagtgtttatcaccaatctttagatgacattcaagacttctatgaagacaaacaattgacga

ttgattttgatatcactggagaaaatcaaattaatcgtggtcccatagacgtcacgttcgagaaatggtg

ggataaccaactgtccaacaacaacacgattggccattatcgaattggcggaatgttcattgaattctca

cgaagcaatgcagctactgtggctagtgaaatagctcacagttctgaacgtgagttcttagtccgtggag

ctgttggtagtggcaagtcaacaaaccttccgtttctgcttagcaaacatggcagtgtgttgttaataga

acctactcgacccctttgcgagaacgtttgtaagcaattacgcggtgaaccgttccattgtaatccaacc

attcgcatgcgtgggctaacggcttttggttctactaacatcacgatcatgacaagtggatttgccttgc

attactatgctcacaacatccaacaattgagactctttgatttcataatctttgatgaatgtcacgttat

agacagccaagccatggctttctattgtttaatggaaggaaatgcaattgaaaagaagatccttaaagtt

tctgccacgccacctgggcgtgaagttgagttttcaacacagttcccaacgaagattgtgactgagcaat

ctatcagttttaaacagctggtagacaattttggcactggtgcgaatagtgacgtaactgttttcgcaga

caatatactggtttatgttgcaagctataatgaggtagaccaattaagtaagcttttatctgacaaaggc

tacttagtcactaaaattgatgggagaacgatgaaagttggaaaaactgaaatttccactagtggtacaa

agtccaagaagcatttcatagttgcaacaaacatcattgagaatggcgtcacacttgatattgaagctgt

catagattttggaatgaaagttgtacctgaaatggactcagacaatcgcatgattagatactcaaagcaa

gccatcagctttggggaaaggattcaaagacttggtcgtgtgggaagacacaaagaggggattgcactaa

gaattggacatacagagaaaggcatccaagaaattccggaaatggcagccactgaagcggctttcctgag

cttcacatatggcttacctgtcatgactcataatgtgggattaagtttactcaaaaactgcactgtacgg

caagcacgcacaatgcagcagtatgaattaagtccattcttcatgcaaaatttagtgaactttgatggca

cggtgcaccccaaaattgatgtgctgttacgtccttacaaattgagagactgtgaggtcagattgagtga

agcagcgataccacatggagtgcaatccatatggatgtctgctcaggaatatgaggcagttggaggccgc

ctttgcctagagggtgatgtgcgcataccatttctcattaaggatgtgcctgagcgattatataaagaat

tgtgggacgttgtacaaacttacaagcgtgactttacatttggccgaattagttctgtgtctgctgggaa

aattgcgtacacattaagaactgatgtgtattctattcctagaactctcataacaattgacaagctgatt

gagagtgaaaacatgaagcatgctcacttcaaagctatgacaagttgcactggtcttaattctagtttct

ctctccttggtgttataaacaccattcagagtagatacttggttgatcattcggttgagaacattagaaa

gcttcacttggcaaaggcccaaattcaacaacttgaagctcatgtgcaagaaaacaatattgaaagtttg

attcaatctcttggagctgtgagagccgtctatcatcaaggtgttgacggagttaagcacataaaacaag

cgttgggcttgaaaggaatttgggacggctcattaatgattaaggatgcgctcgtatgtggttttacaat

ggctggtggtgcaatgcttttgtatcaacactttcgtgacaagctcacaaatgttcacgtgttccatcaa

ggtttttctgcgcgacaacgacaaaagctaagatttaggtctgctgcacatgctaagcttggccgagaag

tttatggagatgatggaacgattgaacattatttcggagaagcatacacgaagaaaggaaacaagaaagg

aaagatgcatggcatgggtgttaagacaagaaagttcgttgcaacatacggattcaaaccagaggactac

tcgtatgttcggtatctagatcctctgacaggagagacactggatgagagtccgcagactgacatctcaa

tggtgcaagagcatttcggtgacatacgaaacaagtacatggaatcagacaactttgataggcaagctct

gatagcaaacaacacaattaaagcttactacatccgaaattctgcgaaaacagcattggaagttgatttg

acgccacacaaccctttgaaagtttgtgacaacaaattaacaattgcaggatttcctgaaagggaagcgg

agctgaggcaaacaggcccacccaagactatccaggctgatcaggttccaccaccatctaaatctgtcca

tcacgaaggaaaaagtctttgccaaggtatgaggaactacaatggcatagcttctgtggtatgccatctg

aaaaacacatcagggaaagggaagagtctatttggggtcggatataattcattcattatcacaaaccgac

acttattcaaggagaataatggtgagcttatagtgaaatcccaacacgggaaatttgttgtcaagaatac

cacaacactccgacttgctccaattgggaagactgaccttctgatcatccgaatgccaaaagatttccca

ccattccatagtagagctaggttcagggccatgaaagctggggacaaagtttgcatgataggtgttgact

accaagagaatcacattgcgagcaaagtatctgagacctctatcattagtgaaggctcaggagaatttgg

atgtcattggatatccacaaatgatggtgattgtggtaatccacttgttagcgtctctgatggctacatt

gtgggactccatagtctgtcaacgtcaactggcgaccagaatttctttgccaaaatacctgcattctttg

aagaaaatgtccttagaaaaattgatgaattaacttggagcaagcactggagttataacattaatgagtt

gagttggggggcattgaaggtgtgggaaagtcggcctgaagcaatctttaacgcacagaaagagatcaat

cagttgaatgtttttgaacaaagtggcagtcgctggctttttgacaagttgcatggtaacttgaaaggca

ttagctccgcacccagcaacctagtgacgaagcatgttgtgaaaggcatctgtcctctctttaagcatta

tctcgagtgtgatgaagaggccaagaccttcttcagtccacttatgggccattatatgaagagcattttg

agtaaggaagcttacacgaaagatttgttgaaatactcaagcgacataattgttggagaggttgattatg

atgtcttcgaagatagtgttgcgcaagttatggacttgttgaatgagcatgaatgccctgaactagagta

catcacagatagtgaagtgataatccaagcactaaacatggatgcagctgttggggccttgtatacagga

aagaagaagaaatattttgaaggatcaacattggaatataggaaagccctcgttcagaagagttgtgaac

gcctttatggagggctaatgggcgtctggaatggctcattaaaggccgaactaaggccagctgaaaaagt

tcttgcgaagaagacaagatccttcactgcagcacctcttgacacattacttggagccaaagtctgtgtt

gatgatttcaacaactggttttatagtaagaatatggagtgtccgtggactgttggaatgacaaagtttt

ataagggttgggatgaattcttaagaaaattcccggatggctgggtttactgtgatgctgatggatctca

atttgatagctcattaacaccctacttgctgaatgccgtcttgtcaattcgattatgggccatggaagat

tgggatattggggaacagatgctcaagaacttgtatggggagatcacatacacacccattttaacgccag

atgggacaatagttaagaagttcaaaggaaataatagtggccagccttcaacagttgttgacaacacatt

gatggttttaatcacaatgtattacgcattgcgtaaagctggttatgatgcaaaagctcagggtgagatg

tgtgtgttctacattaacggtgatgatctctgtatcgccattcacccggatcacgagcatgttctggact

cattctctaactcatttgctgagttaggacttaagtatgattttactcaaaggcatcgaaataagcagga

tttatggtttatgtcgcatcgaggtgttctgatcgatgacatttacattcccaaacttgaacctgagagg

atcgtggcaatccttgaatgggataaatccaagctcccagagcacagactggaagcaatcacagcagctt

tgatagaatcatggggttacgaagagctaacatatcaaattcgtagattttatcgatgggttcttgaaca

agctccattcaatgaactggcaagacaaggcaaggctccttatgtctctgaagttggtcttagaaggttg

tacactagtgaacgtggaacaattgacgaactagaagcttacatagataaatactttgagcgtgaaaaag

gagattcacctgaactacttgtgcaccatgaatcaagtagtgttgcaataaagaatcaatttttgtgcgg

tagcgacaagcacgtctaccatcagtcgaaagctgaggctgtggatgcgggcttaaacgataagctcaaa

gagaaagaacaaaaagaaaaagagaaaaagaaagaaaaagacgaagctggtggcggaaatgatgtgtcaa

ccagcacgaaaactggagagagagatagagatgttaacgctggaaccagtggaactttcacagttccaag

aataaagtcatttactgacaagatggttttgccaagaattaagggaaaaactgtccttaatttaaatcac

cttcttcagtataatccacagcaaattgacatctctaacactcgtgccacccaatctcagtttgagagat

ggtacgagggggtgaggaatgattatggtcttaatgatatcgaaatgcaagtgatgttaaatggcttgat

ggtgtggtgcatcgaaaatgggacatctccggacatatctggtgtctgggtaatgatggatggcgaaact

caaatcgaatatccaatcaagcctttgattgaacatgcaactccctcatttaggcagatcatggctcact

tcagcaacgcggcagaagcatacattgcaaagagaaacgcaactgaaaagtacatgccgcggtatggaat

caagagaaatttgactgacactagcctcgctagatatgctttcgatttctatgaggtgaattcgaaaacg

cctgatagagctcgcgaagctcacatgcagatgaaagctgcagcgctacgcaacgctaatcgtagaatgt

ttggcatggatggtagtgtcagtaacaaggaagaaaacacggagagacacacagtggaagatgtcaacag

agacatgcactctctcctgggtatgcgtaactgaatacccacgcttgtgtgttcgtcaggcctggcttga

ctctgttttaccttatagtactatttaagcattaaaatatagtgtggcttcgccacctttcgtattttat

agtgagggcatccctccgtgcttttagtattattcgagttctctgagtctccatacagtgtgggtggccc

acgtgctattcgagcctcttagaatgagag
